# Foraging in Boreal Forest: Wild Food Plants of the Republic of Karelia, NW Russia

**DOI:** 10.3390/foods9081015

**Published:** 2020-07-29

**Authors:** Valeria Kolosova, Olga Belichenko, Alexandra Rodionova, Denis Melnikov, Renata Sõukand

**Affiliations:** 1 Department of Environmental Sciences, Informatics and Statistics, Ca’ Foscari University of Venice, Via Torino 155, 30172 Venice, Italy; valeriia.kolosova@unive.it; 2 Institute for Linguistic Studies, Russian Academy of Sciences, Tuchkov pereulok 9, 199004 St Petersburg, Russia; 3 Institute of Linguistics, Literature and History of the Karelian Research Centre, Russian Academy of Sciences, Pushkinskaya St. 11, 185910 Petrozavodsk, Russia; santrar@krc.karelia.ru; 4 Komarov Botanical Institute, Russian Academy of Sciences, Professor Popov St. 2, 197376 St Petersburg, Russia; DMelnikov@binran.ru

**Keywords:** ethnobotany, Karelia, wild food plants, Local Ecological Knowledge, Nordic studies, boreal forest

## Abstract

While the current consumption of wild food plants in the taiga of the American continent is a relatively well-researched phenomenon, the European taiga area is heavily underrepresented in the scientific literature. The region is important due to its distinctive ecological conditions with restricted seasonal availability of wild plants. During an ethnobotanical field study conducted in 2018–2019, 73 people from ten settlements in the Republic of Karelia were interviewed. In addition, we conducted historical data analysis and ethnographical source analysis. The most widely consumed wild food plants are forest berries (three *Vaccinium* species, and *Rubus chamaemorus*), sap-yielding *Betula* and acidic *Rumex*. While throughout the lifetime of the interviewees the list of used plants did not change considerably, the ways in which they are processed and stored underwent several stages in function of centrally available goods, people’s welfare, technical progress, and ideas about the harm and benefit of various products and technological processes. Differences in the food use of wild plants among different ethnic groups living in the region were on the individual level, while all groups exhibited high variability in the methods of preparation of most used berries. The sustainability of berry use over time has both ecological and economical factors.

## 1. Introduction

Different ethnic and linguistic groups sharing the same territory do not always use the same wild edibles in similar ways [1,2,3]. While the current consumption of wild food plants in boreal forests on the American continent is a relatively well-researched phenomenon (see, for example, [4] and references therein), the European taiga area, especially the European part of the Russian taiga, is heavily underrepresented in the scientific literature, both recently as well as historically. Nowadays, collecting wild food plants in the taiga fulfills some additional functions, being a recreational activity and part of the healthy diet of contemporary city dwellers who are concerned about ecologically friendly production, depending on the economic situation of the region and specific person [5,6,7]. While there has been a comprehensive review of the historical and current uses of wild food plants listed in Russian pharmacopeia [8], ethnobotanical publications on the Russian Federation based on modern field studies are still few in number (but see, for example, [9,10,11,12,13]). 

The common narrative one encounters in Russia is that wild food plants have always been an important addition to the diet of peasants in pre-modern societies, especially in the North, where the climate across most of the territory makes agriculture rather unsustainable [14,15]. However, according to pre-revolution Russian statistical tradition, welfare was mainly estimated by the consumption of bread; for instance, in a book devoted to the food of the Russian peasantry [16], mushrooms and berries are not mentioned. Nevertheless, sporadic data can be extracted from the ethnographical literature. A.G. Gudkov analyzed the phenomenon of exporting corn from the Russian North (Vologda, Olonets and Arkhangel’sk gubernias) in spite of regular crop failure. Bread shortage from regular arable land was compensated for by natural food reserves: fish, game, mushrooms, berries, and wild bread substitutes. In the 18th–19th centuries, during lean years, the cambium of *Pinus* was added to flour for making bread. The other bread substitute consisted of a group of wetland plants; a tradition definitely borrowed by Russian peasants from the aboriginal Finno-Ugric population around the 13th century [17].

(The Republic of) Karelia is characterized by limited agriculture due to its northern location and mostly forested landscape and thus a higher percentage of wild food (as products of fishing, hunting, and gathering) in the daily diet. The ethnobotanical knowledge of the Karelian region has not been described or analyzed to date. The works by Lebedeva and Tkachenko [10,18,19] that mention Karelians were based on published sources and primarily concern Karelians living in other parts of NW Russia—Leningrad, Vologda and Tver’ regions—and are solely quantitative, paying little attention to detail, such as preparation methods and foods made, time of use, etc. 

Karelians, as all peoples of the former USSR, exhibited the tendency to level and erase zonal differences in food, bridging urban and rural foods [20]. While until WWII numerous features of traditional cuisine were preserved in Karelian villages, evacuations during the war, eateries in forest villages and state farms, and everyday communication with all the different ethnic groups living in Karelia contributed to the exchange of culinary knowledge and affected the formation of modern Karelian cuisine. New food products and dishes came into everyday life. Finally, the increase in well-being, the development of gasification and electrification, and the introduction of household appliances contributed to a significant improvement in nutrition, increased calorie content of cooked dishes and their variety, better preservation of products, etc. [21]. However, “a number of national dishes still occupied, in the final years of the Soviet Union, a prominent place in the diet of the Karelian population” [22]. At the end of the Soviet era, a book was published about Karelian cuisine [21], which was re-printed several times and became very popular, being kept in many households, including those of our informants. Unfortunately, this edition included not only recipes of both traditional and contemporary cuisine (in cities and the countryside) but also recipes from other books, Vepsian and Finnish dishes, as well as the specialties of catering enterprises in Karelia, not always indicating the source of the information. 

The aims of the current work are:

- to document the food plants used in the Karelian Republic among Karelians and Russians, as well as temporal changes in their collection, storage, and use;
- to compare the uses between different groups and within the lifetime of the interviewees; and
- to evaluate the possible sources of, or influences on, differences in uses and changes during this time period.

## 2. Data and methods

### 2.1. Study Area

**Figure 1:**
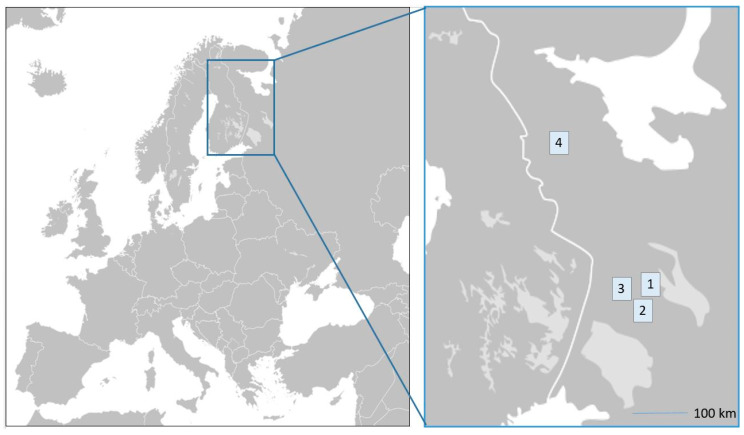
List of fieldwork sites: (1) Petrozavodsk, Zaozer’e, Lekhnavolok, Novaya Vilga; (2) Priazha; (3) Essoila, Korza, Rubchoila, Siamozero; (4) Kalevala. Map base: http://upload.wikimedia.org/wikipedia/commons/5/5a/BlankMap-Europe-v4.png.

The Republic of Karelia is a region in NW Russia (180,500 sq. km) bordering Finland. According to physico-geographical zoning, Karelia is included in Fennoscandia, occupying its southeastern part [23]. Eighty-five percent of the territory is composed of state forest stock; and it is home to Ladoga and Onega lakes—the largest in Europe. The case study site is characterized by mostly rural settlement (Figure 1), low population density, and free access to forests and wild plants, as well as hunting and fishing. 

The contemporary ethnic picture of the region is the result of long processes of economic and political change. In the 1930s–1950s in the USSR, within the framework of the state’s repressive policy, repeated mass forced displacements of prisoners were carried out to create the workforce needed for the development of natural resources necessary for industrialization. For instance, in 1937 in the Karelian Autonomous Soviet Socialist Republic, 28,130 political prisoners were forced to work at the Belomor-Baltic factory of the NKVD [Rus. *Narodnyi Komissariat Vnutrennikh Del*/People’s Commissariat for Internal Affairs] [24].

In the 1940s, in the southern part of the region—the Karelian Isthmus and Northern Ladoga—was depopulated and repopulated twice. Finns abandoned their homes on two occasions, and both times their houses were occupied by Soviet migrants, who first appeared in these lands in 1940, after the USSR annexed the territory from Finland. But a year later, in the summer of 1941, Finland, supported by German troops, regained the territory, and thus the Soviet residents were hastily evacuated. A significant portion of the former Finnish population returned to the Karelian Isthmus and Ladoga region, yet they were again forced to leave their homes in 1944 due to the advance of Soviet troops. A resettlement campaign was then again launched in the USSR, which included the return of evacuated immigrants of 1940, as well as many new people arriving from regions badly damaged during WWII. As a result, by the 1950s, several ethnic groups were living together in a relatively small area: Russians, Belarusians, Ukrainians, Chuvashs, Tatars, Mordovians, Ingrian Finns and others [25,26]. By 2002, Karelia had become a multinational republic with a predominance of Russians. There are currently about 150 nationalities represented in total, including 548,941 Russians, 65,651 Karelians, 37,681 Belarusians, 19,248 Ukrainians, and 14,156 and 4870 Finns and Veps, respectively [27].

The Karelian language belongs to the Baltic-Finnish branch of the Finno-Ugric language family. Throughout the territory, the Karelian language is divided into three main dialects—Karelian Proper, Livvi, and Ludic—as well as smaller dialects within each. Karelian Proper is widespread in the central and northern parts of the republic, in the territory of present-day Kaleval’skii, Loukhskii, Belomorskii, Kemskii, Medvezh’egorskii, Muezerskii and Suoiarvskii districts. The Karelian Proper dialect is also spoken in the regions of Tver’ and Leningrad. The Livvi dialect is widespread on the northeast coast of Lake Ladoga (southern regions of Karelia). Ludic Karelians traditionally live in a number of villages and towns in the southeastern part of the Republic of Karelia in Olonetskii, Priazhinskii and Kondopozhskii regions. The Ludic dialect is an intermediate link between the Livvik dialect and the Veps language [28]. From the point of view of Finnish linguists, “Karelian is divided into two (or three) main dialects, which are sometimes referred to as separate languages: Karelian Proper, consisting of North (White Sea) and South Karelian dialects, and Olonets Karelian (Olonec Karelian, Olonetsian, Livvian)”, with Ludic as a separate language [29].

In the Republic of Karelia, Karelian is an autochthonous language, based on the Latin alphabet, which does not have the status of an official language; and the number of speakers has been steadily declining. According to official census data, in 2002 there were 65,651 Karelians, which amounted to 9.2% of the population, which dropped to 45,570, or 7.4%, in 2010 (see Table 1, and [30]). “All dialects of the Karelian language spoken in Russia are under serious threat of extinction. <...> According to the 2010 census, the number of those who consider themselves Karelian and those who have some competence in the Karelian language decreased sharply in just eight years. Now those who indicated “Karelian” in the column “nationality” have become 34.8% less, and only half of them indicated that they know the Karelian language” [31].

There are TV and radio programs broadcast in Karelian by the local state teleradio company *Karelia* (http://tv-karelia.ru/?id=14648). There are also two weekly newspapers, Vienan Karjala [Belomorsk Karelia] (in Karelian Proper), and Oma Mua [Own Land] (in Livvik). In these media outlets, “children are seen as the future of the Karelian language. Therefore, great importance is given to education, and implicitly also to politicians and state officials who decide on the sharing of resources” [34].

The prospects of revitalization of the Karelian language have recently been described as bleak [29], but also encouraging based on the teaching of Karelian at schools and pre-schools [35,36]. For example, in Vedlozero, there is the so-called “language nest”, a group of children of pre-school age organized as a kindergarten in the Karelian language, which has been open since 2017 [37].

### 2.2. Fieldwork

In the summer of 2018 and 2019, we visited nine villages and urban-type settlements, as well as the capital city of Petrozavodsk, in the Republic of Karelia, where we interviewed 73 people, including 12 men and 61 women. The oldest respondents were born in 1927, while the youngest was born in 1986. We used the snowball method, asking inhabitants about their oldest and most knowledgeable neighbors.

The language of the interviews was Russian, which is spoken by everyone in the region. The conversation began with free listing, followed by semi-structured interviews, which consisted of questions about the use of wild edibles in foods such as soups, pies, jams and other desserts, spices, salads, snacks, roots, recreational teas, and other drinks, as well as plants for smoking meat and fish. After the interviews, we requested permission to take some dry specimens from our interviewees’ winter stores of dried herbs and conducted field walks when the weather and the health of the person permitted in order to collect voucher specimens.

The Plant List database (2019) [38] was used as the basis for plant nomenclature. The botanical families were classified according to the Angiosperm Phylogeny Website [39]. The herbarium specimens are located in the Herbarium of the Komarov Botanical Institute, Russian Academy of Sciences, Saint Petersburg (LE), bearing accession numbers LE 01063338-91, LE 01063417-20, LE 01063464, LE 01063479-95, LE 01063497, LE 01063499-503, LE 01063507-09, LE 01063512-13, and LE 01063533-40.

Before the interviews, we explained the aims of the project to our respondents and received their oral or written consent for the interview and/or using an audio recording device. The study was conducted in accordance with the ethical guidelines of the International Society of Ethnobiology [40]. Ethical approval was granted by the Ethics Committee of Università Ca’ Foscari; while the fieldwork was supported by the Institute for Linguistic Studies, Russian Academy of Sciences.

Responses were transcribed from the recordings or in rare cases from notebooks, and subsequently entered into an Excel spreadsheet according to emic Detailed Use Records (DUR, the number of use records considering all details of use, e.g., the plant part and specific preparation involved *sensu* [41]). As wild food plants, we considered all plants used for food that grow without direct human involvement, including those naturalized or those cultivated for non-food proposes [42].

Karelian phytonyms were recorded and used to qualitatively evaluate the level of phytonymical knowledge of the Karelian population. Romanization of the Cyrillic script of the original Russian words was done according to the ALA-LC (American Library Association—Library of Congress) Romanization without Diacritics set of standards (https://www.convertcyrillic.com/#/).

As to illustrate our interpretations, we use quotes from the anonymized interviews with the study participants. In all such cases, we use a reference identifying the particular interview among others indicating gender, ethnic group and the age of the participant in brackets following immediately after a quote, for example (F, Karelian, b. 1934).

The participants were first selected using the convenient selection technique, although later the snowball method was also sometimes used. The main criterion for participation in the study was the claim that the person was local, which was the case at the start of all interviews, as we aimed to interview an equal number of Karelians and Russians. However, some of our interviewees revealed by the end of the interview that they had actually been born elsewhere or were from mixed families. In order to make a sensible comparison, we decided to form three groups to be compared in Table 2: 

Karelian—both mother and father speak Karelian; 

Russian—originating from nearby villages with both parents Russian-speaking; and

Other—representing other ethnic groups of the region (e.g., Veps, Finns, Inkeri), as well as migrant Russians, Belarusians, Ukrainians, Poles, and Germans, for whom one parent, or very rarely the person themselves, had re-located in youth. 

For temporal comparison, we developed specific categories, representing the obtained data, dividing the results into past and current uses. 

Past uses (no longer in active use) included:

childhood use only—plant uses learned and abandoned in childhood (circa 1940–1950s);
past use—mainly abandoned a long time ago, predominantly used by the informant’s parents, grandparents, or other older relatives, and less often themselves;
abandoned recently—learned in childhood, yet abandoned up to a few years ago for various reasons;
temporarily used in adulthood.

Current uses included:

always used—more or less continually from childhood to the present;
learned as adult—unknown in childhood and learned as adult;
learned only recently (since circa 2000, but mainly in the last five years).
For visualization of the comparative analysis we used RAWGraphs [43].

### 2.3. Historical Data and Published Data

Presumably the first mention of wild edibles in the territory of modern Karelia can be found in the *Saga of Halfdan* (circa 1230) by Icelandic historian and poet Snorri Sturluson: “After that, you come to the forest which is called Kálfárskógr, it is sixteen by twenty transitions long; there is no food there except berries and wood sap; there is a robber there named Selr, and with him a dog, as big as a bull; it has a human mind and in battle it is better than twelve men” [44]. “Wood sap” (Old Islandic *safa*) in some texts is combined with not only the verb “drink” but also “eat”, which suggests that the noun itself may not only mean tree sap but also cambium. In any case, we have very old written evidence of food foraging in the forests of (future) Karelia.

More detailed descriptions of various berries in the food of the local population, as well as some dishes made from these fruits, were provided in 1785 by the famous Russian poet Gavriil Derzhavin [45], who at the time was ruler of the newly formed Olonets governorship, to which the territory of present-day Karelia belonged. Later ethnographic data were taken from books and journals dating from the end of the 18th century to today. The used journals published ethnographic descriptions as well as travel notes. The correspondents were both professionals, like Ivanov (journalist and local historian) and Dokuchaev-Baskov (historian), and just local correspondents (Rogachev, Kalinin, etc.), often publishing under shortened surnames (e.g., Ostr., V-gov) or even just initials (A.A.Zh.), who provided interesting data on the quantity of berries that can be stocked and stored, prices at local markets, etc. Still, most of these publications were descriptive in nature and did not pretend to be scholarly research. That is why they often do not provide many details on cooking or storage; many authors just enumerated berries (and, much more seldom, herbs). 

The first ethnographic description of the city of Onega was written by ethnographer S. Korablev [46]. A more popular description of the Russian North was made by a young writer named S. Maksimov [47] as a result of a “literary expedition” in 1855; a number of these expeditions were organized by the Russian Marine Ministry “to study the life of residents involved in sea affairs and fishing, and to compile the articles in the ‘Marine Collection’” [48] (p. 19). As researchers mostly paid attention to food, houses, clothes, and crafts, it is possible to find some useful information in chapters concerning traditional dishes, such as in a book by K. Loginov about Zaonezh’e [49], based on materials of the ethnographic expeditions. The same researcher, K. Loginov, the leading ethnographer of the Karelian Research Centre, compiled the corresponding chapters into collective monographs [50,51,52], based on published data, archival sources (housed at the Karelian Research Centre), and field interviews. Of particular interest for this study are the works of R. Taroeva (Nikol’skaia) [21,22,53]. Taroeva, who also worked at the Karelian Research Centre, had a special interest in folk cuisine, and thus there is more information about processing wild edibles and preparing various dishes with them in her works, based on interviews conducted during her fieldwork.

We gathered all available data into Table 3; however, we did not consider entries that just mentioned “berries” without any specification. In addition, the cases in which the names of the berries are given, but their uses not described, were also omitted. The presumably scientific literature is very heterogeneous, consisting of recommendations for times of famine, and the majority of the books are partly popular scientific, in which the bibliography is included at the end of each chapter. As the ethnobotanical data was not collected purposefully and only mentioned in passing, in most cases it can only be used as material for comparing the variety of plants used in the past with those of the present. Unfortunately, nearly the same style continued in ethnographic publications during Soviet and post-Soviet times. Therefore, it is not possible to understand specifically where the information about wild edibles was obtained and if it is a local use or a generalization of uses from many regions. As a result, we recommend approaching this data with caution. 

Taking these considerations into account, the Results section is merged with discussion and is split into two major parts. The first part is dedicated to the description of field data and their comparison with the available historical and ethnographical data. The second part is addressing the problem of recommended WEP (wild edible plant) use that became more and more important during Soviet era. Some coincidental practices that served as a favorable context for these recommendations, such as state induced harvesting of the wild edible plants, are discussed in the subsection about the phenomenon of procurement offices.

## 3. Results and Discussion

In Table 2, uses of wild edible plants by our informants are shown. It includes official names and families of the plants, their local names (Karelian names are written only in case they were given by the interviewees, details concerning used parts, preparation, and culinary use. KAR—Karelian, RUS—Russian, OTH—other ethnic groups, see description on p. 5. Numbers before and after slash represent current and past uses respectively.

Comment to the Table 2: *Kissel* (Rus. *kisel’*) is a viscous fruit drink. It consists of the sweetened juice of berries thickened with starch. *Kompot* (Rus. *kompot*) is a non-alcoholic sweet beverage, made by cooking fruit in a large volume of water with sugar, sometimes with various spices. *Kvass* (Rus. *kvas*) is a non-alcoholic beverage made of fermented rye bread. *Mors* (Rus. *mors*) is a fruit drink prepared from berries. It is made by boiling berries with sugar or just mixing pure juice with sweetened water; the berries themselves are then filtered out, to differ from *kompot*. *Mousse* (Rus. *muss*) is a sweet dish made of berry juice, semolina, and sugar syrup. *Okroshka* (Rus. *okroshka*) is a cold soup, a mix of raw vegetables, boiled potatoes, eggs, and a cooked meat (or sausage) with *kvass* (more seldom with *kefir* or mineral water). *Tolokno* (Rus. *tolokno*) is a traditional finely milled mixture made of steamed, dried, lightly fried and refined grains of oats. *Vareniki* (Rus. *vareniki*) are filled dumplings made by wrapping unleavened dough around a savory or sweet filling and cooked in boiling water. 

Of the 70 taxa used throughout the lifetime of our interviewees, 61 were identified at the species level, eight at the genus level and one at the family level (Table 2). Of the 27 families represented, the most numerous were Rosaceae (13 taxa), Asteraceae (seven taxa), Ericaceae (six taxa), and Poaceae (four taxa). Among the ten most commonly used taxa, berries of three *Vaccinium* species dominated, including *Vaccinium vitis-idaea* (280 DUR), *Vaccinium oxycoccos* (199 DUR), and *Vaccinium myrtillus* (194 DUR), followed by *Rubus chamaemorus* (131 DUR), *Betula* (82 DUR), and acidic *Rumex* (60 DUR). Therefore, the most widely used families were Ericaceae (727 DUR), Rosaceae (383 DUR) and Betulaceae (113 DUR). 

**Figure 2:**
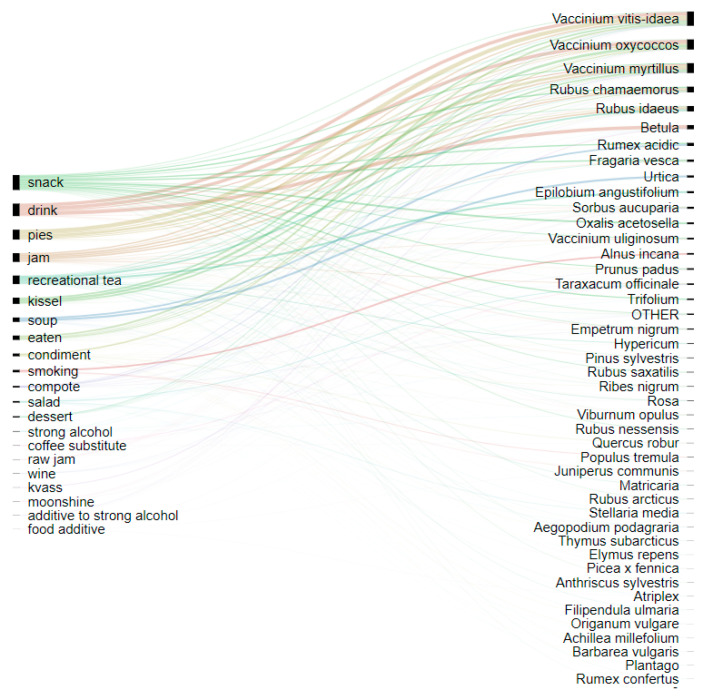
Alluvial diagram showing the relationships between foods made and taxa based on DURs during the lifetime of interviewees.

The most popular food categories were snacks (294 DUR), drinks (245 DUR), pies (194), jam (172), recreational tea (167 DUR), and *kissel* (122 DUR) (Figure 2). Berries are used for cooking most types of dishes. Recreational teas are the only relatively popular group of food that uses non-berry plants. Soups include only two plants—*Rumex acidic* and *Urtica*—which represent the first sources of vitamins in spring. Taste additives are the least popular food products.

The plants that were used as snack in childhood and abandoned later include all edible forest berries, leaves of *Oxalis acetocella*, resin or needles of *Pinus sylvesris*, sweet juice sucked from the stems of various grasses, and nectar of *Trifolium*. Among them, the berries of *Daphne mezereum* were mentioned as tasted and spat out by children, as if to confirm that they were not suitable for eating: “They are so poisonous, if one eats, for example, five or six berries, it is possible… in general, it’s very, very dangerous. I remember that at the beginning… it is bitter, eating it… we spat it out and it was kind of OK [laughs]” (F, Karelian, b. 1948). Of course, the fact that the berry is poisonous was recognized by the adults and communicated to the children. Nevertheless, they tasted it to make sure that the berry was indeed poisonous and to discard it in future. The childhood practice of tasting wild plants, even those that are not edible, is not unique to Karelia. It has also been documented in Estonia [54], while some researchers suggest that the wild plants tasted in childhood could in fact relate to the archaic layer of the same tradition [55]. The same Karelian woman later added that the berries of *D. mezereum* were also used by a local healer to make an ointment that would cure a hernia in a child. To prepare it, the healer would request that the mother of a sick child take a bite of each berry “only if her mouth was okay and her teeth were in good health”, otherwise “grandma herself crushed each little berry on an axe in the doorway”.

Besides being directly eaten, there are a number of plants that are used in cooking but their “role” in the final product is only to add taste. The largest such group consists of trees and bushes used for smoking meat and fish, with the most used taxa being *Alnus incana* and *Populus tremula*. The second group consists of spices. Most of the population of Karelia never used spices bought from shops as they were very expensive and thus available only to wealthy people: “Seasonings, besides pepper and onion, are unknown, as is vinegar. Tea is drunk by the rich on Sundays” [14]. This tradition turned out to be so sustainable that even during Soviet times, when seasoning dishes became possible, many housewives did without them. The exception being fresh fish soup. In fact, only bay leaves and black pepper were discussed in the abovementioned book [21]. Perhaps this habit of not using seasoning has also influenced the minimalism in spice use today—the spicy herbs mentioned by our respondents were not very numerous.

**Figure 3:**
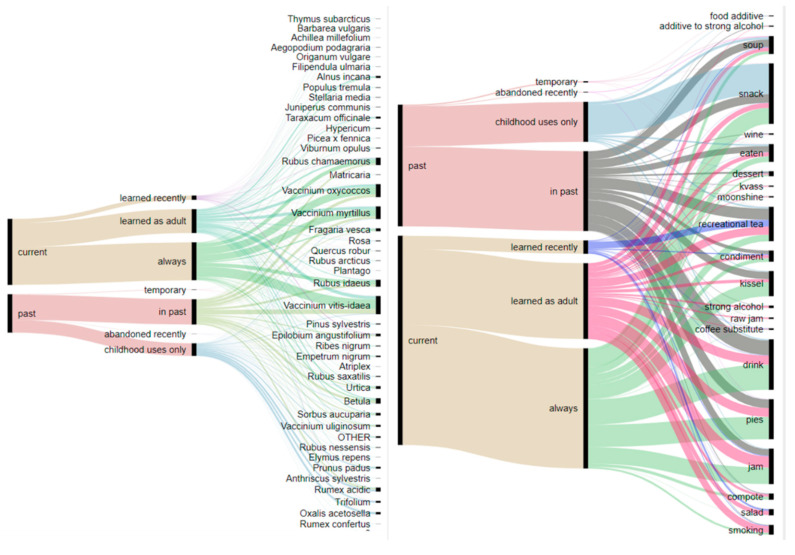
Alluvial diagram reflecting changes in use during the lifetime of interviewees.

As shown in Figure 3, the use of wild food seems to be stable, although having changed several decades ago, with very few species acquired recently or used temporarily. The largest category is permanent uses, while the numbers of taxa acquired in adulthood or abandoned in the past are also quite considerable. The majority of taxa, and in particular berries (*Vaccinium*, *Rubus chamaemorus* and *Rubus vitis-idaeus*), are used all the time. The use of *Betula* sap varies between informants: some have always been using it, others used it in the past, while still others acquired it in adulthood. *Rumex acidic* and *Oxalis acetosella* are used as snacks in childhood and later abandoned. 

The tradition of adding wild greens to soups represents mostly a past use. The category of recreational teas is the most fluid due to opposing tendencies: there is no need to substitute currently widely available, commercially produced tea, yet at the same time users stick to the herbal infusions known since childhood and widen their repertoires by adding new wild species like *Epilobium angustifolium*. Strong alcohol is currently made by very few people, and only two interviewees mentioned it in connection with the past.

With respect to changes that are not formally reflected in Table 2, our informants primarily noted a sharp decrease in the quantity of stored products, caused by a decrease in the number of family members: “Cowberries are mainly [stored] by us. Cowberries are collected in autumn, in a barrel. Oh, twelve, ten buckets each—such big barrels. And everything was consumed during the winter. That’s how many vitamins people ate! And now, three three-liter jars will collect—‘Oh, I’ve got a lot of cowberries!’ Well, now families are... Earlier, the families were not one or two people” (F, Karelian, b. 1934). Another reason for the decrease in stored products is that many informants have moved from large village houses with a basement, attic, and many outbuildings to comfortable, but small flats in urban settlements: “You know, I started to cook *mors*, because there are no conditions to store *kompot*. My sister cooks *kompot*, keeps it in the basement. I have no place to store it, that’s why I put berries in the freezer” (F, Karelian, b. 1960).

Elderly people have to buy berries and mushrooms since they are not able to go to the forest; and some middle-aged people do the same, as their work does not allow them to spend time collecting forest products. On the other hand, unemployment and low salaries compel a part of the population to consider mass harvesting wild plants as a way of generating income, which leads to the over-harvesting of berries and mushrooms. Recently, the increasing levels of deforestation and bears coming into villages hinder the collection of mushrooms, berries and herbs, both for sale and for personal use. Field studies show the same tendency, to be precise, a wave-like decrease in the yield of cranberries even in the most abundant berry-bearing bog areas of Karelia from 2000 to the present. The decline in crop yields is likely due to global climate change: unstable weather conditions during the autumn-winter period negatively affects the formation and wintering of cranberry flower buds [56].

Some people remembered garden bushes and other cultural plants which became wild after Finns left their homes: “There were Finnish farms on the border—there I saw white currants for the first time. They are as sweet as sugar, unlike red and black ones. <...> The first time I saw rhubarb was in the forest, because there were Finnish farms along the border. They were demolished, but the gardens remained. You go through the forest—currants, rhubarb, just thickets. Wild, after the Second World War. In some places, even hops grew in the forest” (M, Russian, b. 1959).

Socio-economic changes in Soviet/Russian society also contributed to changing the set of plant foods, both cultivated (cabbage, cucumbers, tomatoes, etc.) and purchased (fruit, spices). Even the simplest vegetables were not grown until after WWII: “Newcomers became [numerous]. They brought this fashion, cabbage to us; I remember that one came from Molotov region [Perm region in 1940–1957]; our old man got married there. And he brought his wife here from Molotov region. And she was the first to plant cabbages in our village. She even baked bread, she placed it on cabbage leaves—and into the oven” (F, Karelian, b. 1934). Another Russian woman born in 1941 recounted: “Salads appeared, [when] my elder sister married a Ukrainian. They then started living in Petrozavodsk. And gradually it came to us, too. These salads. Tomatoes, cucumbers, all that; people passed [knowledge] to each other”. 

The methods of processing and storing products have changed as well. So, drying berries, soaking them in water, and storing them in the shed in winter time have given way to storage in freezers, even in villages. Access to sugar in unlimited quantities led to the mass production of jams, which subsequently declined due to the belief in the impact of sugar on the incidence of diabetes: “People did not make jam, because sugar was needed. After all, why was it crushed—there wasn’t as much sugar as now. Sugar is a harmful product. People began to live well, so there is a lot of diabetes” (F, Rus/Kar, b. 1948). Homemade pickling, popular in the 1980s–1990s, became limited due to the fear of botulism. Juice cookers and meat grinders with special nozzles are used for making home-made juices and pitted jams, and blenders to make smoothies: “We like making green cocktails and smoothies. It’s 70% of any greens, and the rest consists of berries, fruits, vegetables—whatever you want to put in it. Then into a blender, add water, mix, add honey or sugar—and drink” (F, Russian, b. 1986). 

**Figure 4:**
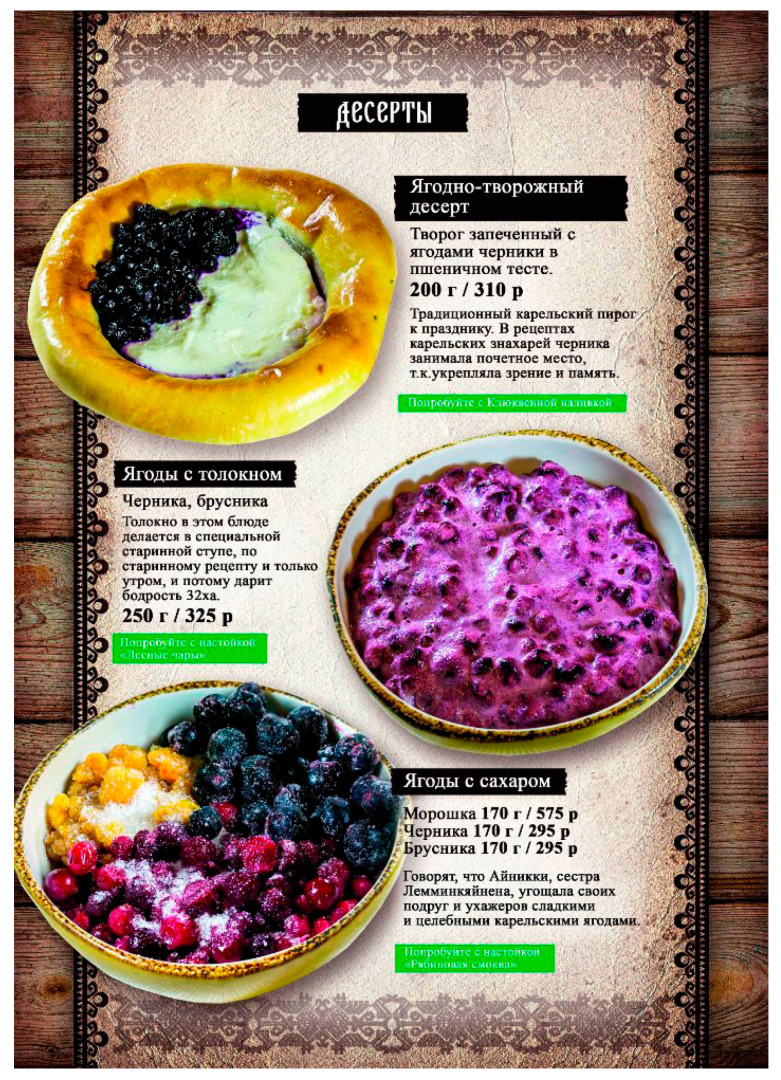
A page from the menu of the restaurant Karel’skaia gornitsa, Petrozavodsk. The second item on the menu: ‘Berries mixed with *tolokno*. Bilberries, cowberries. *Tolokno* for this dish is prepared in a special mortar following an old recipe and only in the mornings so that it good at producing high spirits’.

Urbanization affected the production of traditional dishes. For example, cooking *kissel* requires starch, which was previously made by country dwellers themselves from their own potatoes: “For us in Karelian village, *kissel* was boiled with potato starch. To eat with a spoon. Starch was self-made. Digging potatoes was over, the biggest was chosen—ugly, one may say. Washed well. I remember; it was not peeled, but grated, rinsed with water several times, the water poured away, there was such a white mass. Dried it, and got potato starch” (F, Karelian, b. 1951). Now starch is bought in the store, but complaints have been raised about its quality. The same applies to the traditional dish “cowberry with *tolokno*”, which is even included in the menu of local restaurants (Figure 4). In the past, *tolokno* used to be made at home. Now cereals are not grown on private farms, and the *tolokno* sold in shops is not in demand due to its low quality: “Yes, yes, not edible. I’ve bought some; one day I wanted cowberry with *tolokno* so badly; my mother-in-law made it all the time, and little children ate it, children were fed with it. And it still remains, I don’t use it. That is how bad the quality is” (F, Russian, b. 1957).

Smoking appeared, according to different informants, about 20 to 40 years ago, but it did not become a method of fish or meat preservation and storage for a long time; it was rather perceived as a way of pleasantly spending free time: “My parents never smoked fish. We mostly salted it” (F, Karelian, b. 1968). Elderly people even said that they didn’t know how to do it: “My son smokes, we don’t, we cannot… And earlier, in Syssoila, I don’t remember smoking” (F, Karelian, b. 1944). Those who now do it, describe it as a non-serious pastime: “Fish wasn’t smoked. No, Karelians didn’t smoke. It is only now that I’m messing around with smoking” (M, German, b. 1950). Using *Alnus incana* for fish smoking, like the smoking itself, seems to be a novelty in this region, practiced by newcomers: “No. Maybe; people smoked after us, but at that time it wasn’t used. The easiest methods were salting, boiling and a kind of preservation—cooked for a long-long time, with seasonings—and into jars” (F, Karelian, b. 1954).

The recent fashion for *ivan-chaj* (herbal tea made from *Epilobium angustifolium*; for more details see [57]) has influenced the production of this drink by some respondents themselves for their own consumption and even for sale: “In a jar, my husband kept [leaves] for two weeks without light, then rolled” (F, Karelian, b. 1951). Still, to some people the instructions seem too complicated: “This is common now, all these leaflets, now there’s a lot on the Internet, how to ferment it correctly. But we just dry it” (F, Belorussian, b. 1950). It is easy for innovation to appear in such circumstances: “A few years ago, when we started making tea, we had several blends. And we were the only ones to make complex blends with black tea. That is, people made all kinds of herbs, mixed them, but not with black tea. And we, kind of—why not smoke it? And we smoked it in some way; and so far no one else does it” (F, Russian, b. 1986).

It is interesting how one of our informants strongly denied the use of roots and tree bark by Karelians: “I don’t remember my friends or anyone digging something up, no. I say, people were not hungry… maybe there was shortage, of course, as I recall now, but not to that extent. Karelians did not eat bark—I’m sure of that for some reason. They would salt fish, kill some game in the forest, put snares when they had no rifles—but bark… They write everywhere: “Karelians ate bark”—that’s not true! They [lived] not bad at all… and my husband, he was such a hunter that… we lived in Severodvinsk, and we were never without game” (F, Inkeri/Karelian, b. 1941). It is obvious that for the current population using the bark of trees (although, in fact, this is about cambium, which they do not realize) is viewed not as a usual and neutral practice, but rather as an unfortunate necessity during war time or an offensive assumption. Only one person mentioned the cambium of some tree (she did not remember which one) as her father’s childhood snack: “Dad said, in childhood, they always took a wire, made an incision, removed the bark, and between the wood and the bark there was such a soft white layer, it was the most delicious thing in their childhood” (F, Karelian, b. 1968).

### 3.1. Cross-Cultural Comparison

**Figure 5:**
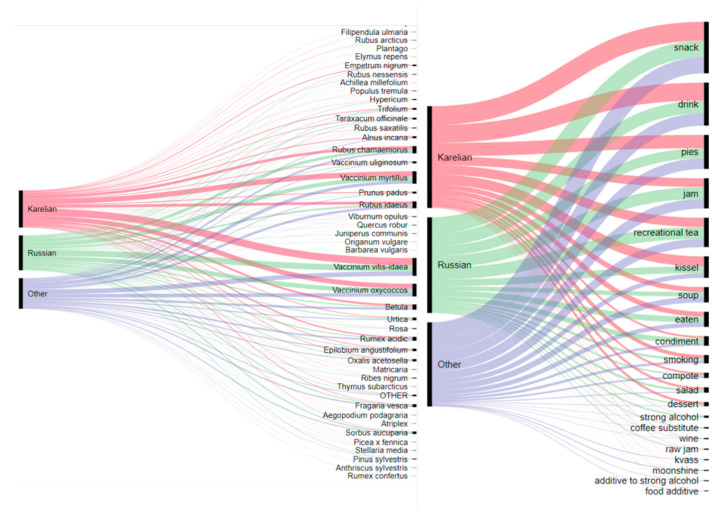
Alluvial diagram illustrating the distribution of plants and uses among the three groups.

Differences in plant use between the different groups appear minimal (Figure 5). The main differences are on the level of the taxa used by only a few people. The majority of plants are used by all three groups, and the differences are observed only with rarely mentioned plants. For Karelians these are *Rubus arcticus, Filipendula ulmaria, Plantago,* and *Elymus repens* (most of them unique to Kalevala, see below), while for Russians they include *Juniperus communis, Origanum vulgare, Barbarea vulgare, Thymus subarcticus* and *Atriplex sp*. For the Other group distinctive plant is *Rumex confertus,* being a childhood snack. The only two food categories not shared by all three groups are “additives to strong alcohol” and “food additives”. The use of strong alcoholic drinks as well as that of condiments are not characteristic of the local culture and have been introduced by newcomers.

**Figure 6:**
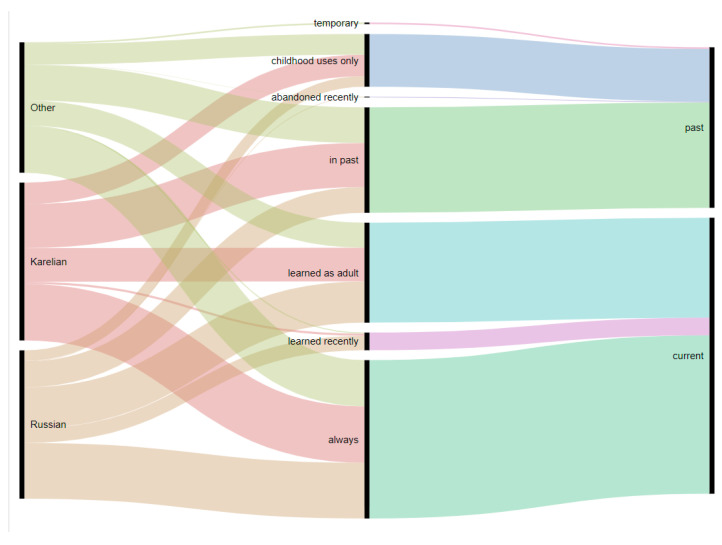
Plant uses in the three groups during the lifetime.

While there is a little difference on the taxa and food levels, we can observe some more pronounced differences on the level of acceptance of recently learned uses. For example, the highest number of new DURs was reported by Russians, who at the same time reported about 40% of uses learned in adulthood and the lowest proportion of uses abandoned in childhood (Figure 6). 

Quite a number of distinctive plant uses come from Kalevala as a result of the remoteness of the region and the cultural and ecological peculiarities of the region related to its isolation. While the use of some plants can be easily explained by characteristics of the habitat (use of the stems of *Eriophorum vaginatum* as a snack in childhood), others are more likely borrowed from the popular literature: for example, the use of *Alchemilla vulgaris* and *Polygonum aviculare* in salads was acquired in the 2000s. The same can be said about the use of flowers of *Filipendula ulmaria* in recreational teas, and the leaves and aerial parts of *Achillea millefolium, Plantago,* and *Atriplex* in salads. One informant mentioned eating the leaves of *Plantago* and various parts of *Elymus repens* in childhood as a snack.

#### 3.1.1. Comparison with Historical Data

Plants and their uses mentioned in the ethnographic literature are given in Table 3. As can be observed, reporting berries as a snack was more the exception than the rule. We can only presume that this was so obvious to the authors that almost no one considered it worth mentioning.

In Table 3, a notable proportion of food uses are represented by additives to flour, mostly the roots of wetland plants. The lichen *Cetraria islandica* was officially recommended by the Russian government in 1841 in a departmental journal which published the following statement: “The Scientific Committee of the State Property Ministry concerned the sample of tree bark used by peasants of Kem’ and Kola counties of Arkhangelsk province to mix with bread, preoccupied with indicating to residents of these counties more suitable substances for this purpose, which could be added to bread and deliver healthier food, and drew attention to Icelandic moss (lichen islandicum) which grows in those lands in great abundance and which, as it is known from the experiments conducted, found fit for food” [68], (p. 258). The following part of the article contained instructions on how to remove the bitter taste from the lichen, dry it, and grind it into flour using ordinary millstones [68]. As there are not references on its actual use for making bread among locals, it may be just a resonance of the campaign of the promotion of the use of wild plants in famine time (see [73]). In historical data, there is no information on making alcohol drinks: “People in Zaonezh’e did not know how to make hoppy drinks of grain, birch sap and aspen bark” [49]; in fact, even wine shops were very few, vodka was seldom sold, and drunkenness was absent [64,74]. In the past, very few herbal plants were used but for flour and tea substitutes—in fact, these are only *Allium*, *Heracleum*, *Pteridium*, *Rumex*, and *Urtica*.

The list of berries used in Karelia, however, did not change for the most part, although in the past they were processed and stored in different and fewer ways. Old techniques are applied to new products: “Karelians use *kissel* a lot: oat and pea *kissel* has been known for a long time, and milk and berry ones were introduced during the Soviet era because of the availability of sugar” [53] (p. 133). At least since the 19th century, local people have collected berries not only for themselves but also for selling in the nearest towns: “In addition to kitchen gardening and fishing, old women-schismatics [Russian Orthodox Old Believers] from Pertozero collect berries, such as raspberries, cloudberries, bilberries (*V. myrtillus*) and cowberries, as well as mushrooms; most of the berries and mushrooms collected are sold to residents of Sumskoi Posad. In recent years raspberries have been sold by schismatics at a rather high price, one might say, i.e., at the St. Petersburg price; so, in the summer of 1909, fresh raspberries were sold at 20 kopecks per pound, while cowberries and cloudberries were sold at 5 to 5.5 kopecks per pound” [61], (p. 15). Some sources provide information about the ways in which to store berries to be sold: “Among berries, fresh bilberries (rarely dried), cranberries and cowberries were harvested for sale. The latter, crushed in barrels with water, were sold to the town, sometimes with soaked wild apples” [50], (p. 106). The extent of the process is underlined by the size of the containers mentioned for the berries: “Cloudberries, soaked in barrels and tubs, were sold to resellers. Fresh bilberries were also bought by them without limitation” [62], (p. 187). 

#### 3.1.2. The Importance of the Name

To evaluate the level of phytonymic knowledge among our Karelian informants, we compared the list of plant names given by our informants in Russian and Karelian with the ones presented in several dictionaries of the Karelian language [75,76,77,78,79,80]. The list of berry names includes 20 in Russian interviews, 12 in Karelian ones, and 19 in the various dictionaries. The ratio for herbal plants is 34:5:29 (but many Russian plant names were named by only one person). In addition, 10 berry names are presented in all six dictionaries while for herbal plants it is only three, which also indirectly indicates the popularity of berries.

For some berries, we were able to record two or three Karelian names, as our respondents speak various dialects of that language. Overall, we had the impression that berry names are retained more by our informants than any other group of phytonyms. They remember mushroom names less, and herb names are remembered least of all: “What’s the name of this plant? [We say *pizhma*] So do we. [And in Karelian?] I don’t know in Karelian. I know all berries names, but not all for flowers” (F, Karelian, b. 1951). One of the informants (F, Karelian, b. 1941) gave the name *juomoi* for *Rubus saxatilis*, although this is really the name of *Vaccinium uliginosum*. Another interviewee gave the Karelian name of *Pinus sylvestris*, i.e., *pedäi*, to *Populus tremula* (F, Karelian, b. 1951). An elderly woman explained her diminishing knowledge of Karelian as a result of living with Russians since the age of fourteen and her marriage to a Russian man (F, Karelian, b. 1927).

In the Russian dialect spoken in Karelia, both *Rumex* and *Oxalis acetosella* may have the name *kislitsa*, based on the sour taste of the two plants (Rus. *kislyi*), as well as *shchavel’*, which is also used for both plants. *Oxalis acetosella* has an especially long list of names which seem to have genesis in children’s culture. The only plant which has special names for soft ripe berries and hard unripe berries in both languages is *Rubus chamaemorus*. 

Sometimes, the plant name was not remembered, yet the person was able to describe it accurately enough for robust identification. For example, we identified *Eriophorum vaginatum* as a childhood snack of a Karelian woman born in 1968: “There are those, they grow in the marshland, plants with white tassels. There are many, many in the swamp… we went to the cinema in the village, and always ate those white ones. Seemed so delicious to us! (...) They are kind of one into another, these little things ... Yes, a blade of grass—you pull it out, and we always ate these tips. They are kind of tasteless, but tasty anyway”.

#### 3.1.3. Food Medicine

To understand the importance of berries to the people of Karelia, it is important to consider, in parallel, their use as medicine: “Again, for the whole *artel’* [a group of workers] one must take—one simply can’t live without it—a barrel of soaked cloudberries; without cloudberries scurvy will lead to death” [47], (pp. 76–77). Raspberries and blueberries [*V. ulginosum*] were dried, and then, as well as cranberries, used for medicinal purposes [22]. Our informants also mentioned *Rubus idaeus* as a means of fighting cold: “My mother always cut the tops of raspberry, twigs with berries, dried them… and then, when she was ill, she brewed these in the tea-pot and drank it as raspberry tea. [Just for taste or as a medicine?] Well, anyway phytoaspirin remains. This fragrance” (F, Karelian, b. 1968); “Raspberry contains natural aspirin” (F, Russian, b. 1948). Other berries were also named as a means to treat various illnesses: “[And high temperature?] Cranberries” (F, Fin/Kar, b. 1941); “Earlier, I remember, my grandmother always crushed cowberries for us. When we had sore throat. And now I make juice for my grandchildren. When there is already a patch there in the throat, tonsillitis, and this acidity—it kills all the germs. I warm it and pour into their mouths. To coat. And then they don’t eat anything for a while. And it goes away very fast” (F, Karelian, b. 1963). After various cold ailments, the second most common problem mentioned was intestinal disorders: “Dried bilberries—for diarrhea. Dries it on the stove” (F, Russian, b. 1938). The same attitude was transferred to cultural plants cultivated in gardens: “Aronia, chokeberry. It also lowers blood pressure, but it increases blood viscosity” (F, Russian, b. 1948); and “I like to harvest viburnum for cough” (F, Russian, b. 1952). It seems that habitual consumption in some cases is thought to have prophylaxis properties: “We should eat berries [bilberries], just fresh berries, they said, for vision” (F, Russian, b. 1967).

### 3.2. Comparison of Current Use with Suggested Wild Food Plants Available in the Region

**Figure 7:**
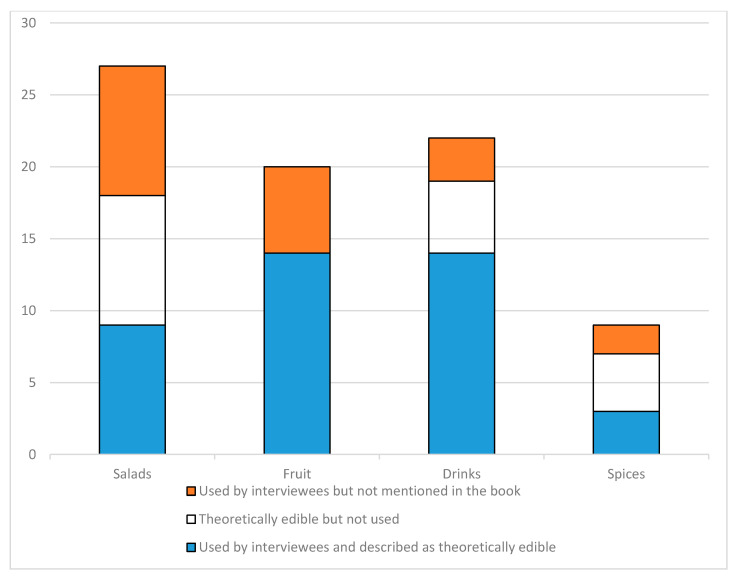
Categories in which theoretically edible wild plants can be used according to [81] and the proportion of the plants recommended in his study against the ones used by our interviewees.

It is possible to compare the situation today with the officially proposed list of edible plants of Karelia [81] (Figure 7). Fruit-yielding plants are used in full (14 out of 14). More than that, during the interviews we encountered plants not listed in the book in question: *Lonicera caerulea*, *Arctostaphylos uva-ursi* and *Vicia cracca* were mentioned only once, whereas collecting *Rubus nessensis*, *Empetrum nigrum*, and *Prunus padus* seems to be common practice. Among the plants recommended for salads only half are used (nine out of 18). Inhabitants of Karelia use nine additional plants, not present in [81], including *Atriplex patula*, *Achillea millefolium*, *Betula* spp., *Stellaria media*, *Trifolium* sp., *Plantago* spp., *Elymus repens*, *Polygonum aviculare*, and *Alchemilla vulgaris*. Still, all of them apart from *Stellaria* were mentioned only once, and in most cases by the same woman who quite actively uses wild edible and medicinal plants. Among the recommended beverage plants, 14 of 19 are used, which includes berries, leaves, and roots, primarily for making *kissel* and *mors*, but also for making tea and coffee substitutes. *Crataegus chlorocarpa*, *Hypericum* spp., and *Betula* spp. (the last as fresh sap, but also to make *kvass*) are also used. Aromatic and spicy plants are not numerous in Karelia, yet still less than half of these are used (three out of seven). Only one of our informants used *Barbarea vulgaris* and *Achillea millefolium*, which are not mentioned in the book.

As we can observe, today all kinds of fruit plants named in the book are used, while salad plants, beverage plants, and spices are underused. It is difficult to judge from only one book, but it appears that here we witness the same tendency toward the maximum use of berries while greens and spices are neglected.

In [55], four groups of wild edible plants in Russia are distinguished: (1) plants harvested and used continuously; (2) plants widely used before but (nearly) forgotten now; (3) plants picked up in spring by children; and (4) plants used during times of war and crop failures. Most of the WEPs mentioned by our respondents correspond to group 1; group 2, based on the above definition, was not mentioned; plants from group 3 are much less numerous than those of group 1; while plants from group 4 were mentioned only by elderly people in reference to memories (their own or those of their parents) about the war and evacuations.

#### Procurement Offices

**Figure 8:**
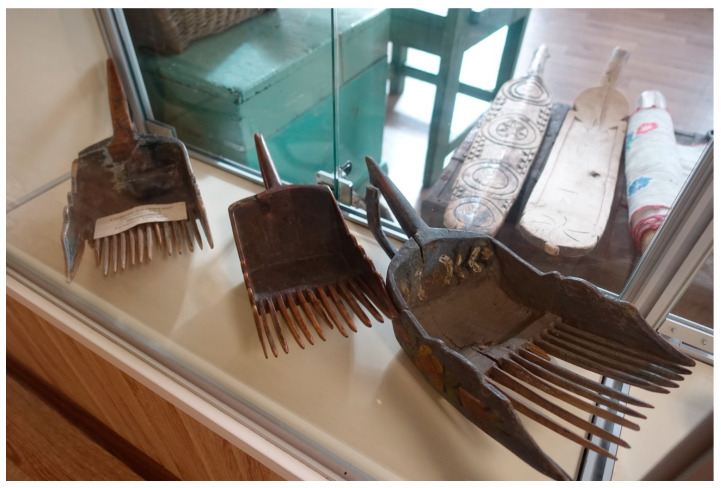
Wooden ‘kombains’ in Ethnographic museum of rune singers, Kalevala.

Another aspect of human-forest relations is that of picking up wild edibles for the procurement office (Rus. *zagotkontora*). By handing over berries, mushrooms, herbs, and bark, people could get not only money but also the right to buy certain goods that were in short supply: “Well, there were procurement offices. There we handed them over—and there were goods. And even imported ones. Finnish goods. Tea, coffee... First of all, berries. Cowberries, bilberries, cloudberries. Blueberries were taken, but they were very few” (F, Karelian, b. 1954). Practically everyone was involved in the business: “Handed over berries, mushrooms, cowberries. Everything was in shortage—clothes, food. One could buy clothes with coupons. We bought imported Finnish clothes. Previously, berries were expensive; it was possible to hand over a lot. <...> I lived in a forest village, and the whole village worked in a timber industry enterprise. And everyone handed them over” (F, Belorussian, b. 1965). The sum of money received for forest products could be rather impressive: “Then real goods, not Chinese fakes, were sold. Japanese electronics, cars. We bought our first car in the procurement office. Had to harvest 500 kg of berries” (F, Russian, b. 1957). The need to collect a large quantity of berries could result in over-harvesting, especially while using special devices for the easy picking of many berries at once (Rus. *kombain*) which was prohibited; however, we saw wooden analogues of such devices made about 100–150 years ago in a local museum (Figure 8). The coupons received for berries and other wild plants sometimes became the object of bargaining: “Or sometimes in a shop, but with a coupon. Suppose, I hand them over for 100 rubles—they give you goods for 10 rubles. For example, the children were small, we could not buy tights. To buy two pairs of tights, it was necessary to hand over a bucket of bilberries. They gave money for it. My husband really didn’t like to collect bilberries, but for four pairs of tights we had to collect. Then these coupons were even outbid by people. People hand them over, who don’t need [the goods], and you pay” (F, Karelian, b. 1954).

**Figure 9:**
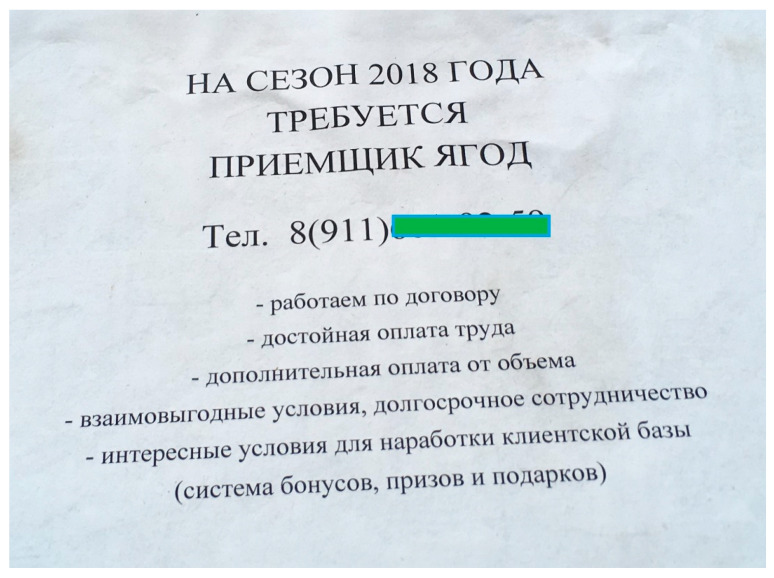
An advertisement about a berry receiving inspector job vacancy for 2018 season, Kalevala. Translation: ‘For the season of 2018 a berry receiving inspector job position is open. Tel. xxx-xx-xx. -job contract, -decent salary; -additional payment in function of volume; -mutually beneficial conditions, long-term collaboration; -attractive conditions for the customer base (a system of bonuses, prizes and gifts)’.

In addition, they could hand over the wild edibles they did not use themselves: “We dried berries, I remember, and handed them over… we needed to buy school textbooks, so we dried and handed them over. But we didn’t use rowan berries” (F, Karelian, b. 1944). In recent years the state offices have been replaced by private harvesters, *ivan-chaj* producers, etc.: “I lived in Kostomuksha before moving here, there was an entrepreneur who took berries and processed them for preserves, juices. And now there is “Iagoda Karelii” [Berry of Karelia]. The whole production is established, and berries are accepted from the population. There, in Kostomuksha” (F, Belorussian, b. 1965). One more way to profit from forest products is to be hired as a berry receiving inspector; this seasonal job is advertised every year (Figure 9).

**Figure 10:**
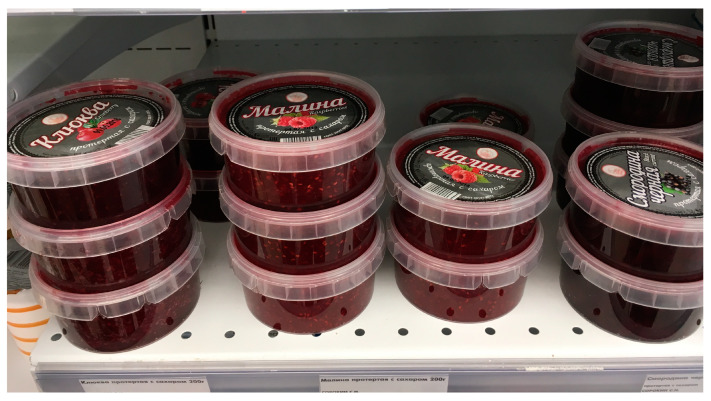
Cranberry, raspberry and black currant jams sold in a convenience store, Petrozavodsk.

‘Iagoda Karelii’ mentioned by the respondent is the first company in Russia to completely process forest berries, from cleaning and sorting to producing fillings, jams, and confitures [82]. Recently, direct investment from Swedish, Finnish and Norwegian companies has become a stimulus for the development of procurement in Karelia. This is due to the proximity of the region to the western borders of Russia and relatively cheap Russian raw materials market. In Karelia, there are about 40 companies collecting wild plants and delivering them to the countries of Northern Europe. All of them work under the condition of full funding from Western partners. Still, the processing of wild-grown berries is not developed in the region: most operators in this market collect berries and export them “as is” [83]. In shops, one can buy jams and other products made from the same berries which have been harvested for centuries (Figure 10).

## 4. Conclusions

As farmers in a risky agricultural zone, the inhabitants of Karelia developed their own type of minimalism, different from, for example, the minimalism of Northern nomads [84]. For centuries, the lack of bread was covered by bread substitutes such as cambium and nutritious wetland plants found in nature, and the lack of fruit and vegetables by berries. In Karelia, changes in traditional culture, usually perceived as the loss of traditional ecological knowledge and plant uses, have actually resulted in the expansion of both the range of plants used (as salad herbs, spices) and the methods of their processing (e.g., smoking, making jam and compotes) and storage (freezing). The only group of plants that has been irretrievably lost is flour additives, which were no longer needed after the problems with bread disappeared. Berries, however, did not disappear after fruit became readily available, and they started to be used in new ways (jams, jellies, smoothies, etc.). This may be explained by both their free availability and their dual role as food and medicinal plants, as well as their being a source of vitamins, especially in winter. Furthermore, in the Russian Empire, in the Soviet Union, and more recently in the Russian Federation, the harvesting of berries was and still is a way to earn extra money and/or obtain deficient goods. 

## Figures and Tables

**Table 1:** Karelians and Karelian speaker population according to the census. The numbers are compiled from [22,32,33].

Year of Census	Karelians in Karelia	Named Karelian as Their Native Language
1989	~79,000	40,685
2002	65,651 (9.2%)	52,880
2010	45,570 (7.4%)	25,605

**Table 2:** Current/past uses of the wild food plants in Karelian Republic.

Plant Taxa	Local Name	Used Part	Preparation	Use	KAR	RUS	OTH
*Atriplex patula* L.; Amaranthaceae	Rus. *lebeda* Kar—	aerial parts	dried	add to flour			/1
			fresh	salad		1	
				soup	1		/1
*Allium* sp.; Amaryllidaceae	Rus. *dikii (zelenyi) luk* Kar—	aerial parts	fresh	soup	/1		
*Aegopodium podagraria* L.; Apiaceae LE 01063539 LE 01063364	Rus. *snyt’* Kar—	aerial parts	dried	condiment for soup		1	
			fresh	salad		2	
				soup		1	1
*Anthriscus sylvestris* (L.) Hoffm.; Apiaceae LE 01063382 LE 01063509	Rus. *dudki* Kar—	buds	fresh	snack		/1	
		stems	fresh	snack		1	/1
*Carum carvi* L.; Apiaceae	Rus. *tmin* Kar—	seeds	dried	spice			/1
*Achillea millefolium* L.; Asteraceae LE 01063537 LE 01063362 LE 01063356 LE 01063417	Rus. *tysiachelistnik* Kar—	aerial parts	dried	spice		1	
			fresh	cooking with fish		1	
		leaves	fresh	salad	1		
*Cichorium intybus* L.; Asteraceae	Rus. *tsikorii* Kar—	root	dried	coffee substitute		/1	
*Cirsium arvense* (L.) Scop.; Asteraceae	Rus. *shchipitsa* Kar—	root	dried	coffee substitute			1
*Matricaria chamomilla* L.; Asteraceae	Rus. *romashka* (*lesnaia)*, *romashka aptechnaia* Kar—	flowers	dried	recreational tea	/2	3	1/1
*Matricaria discoidea* DC.; Asteraceae	Rus. *dushistaia romashka* Kar. päivykukkaine	flowers	fresh	snack		1	
*Taraxacum officinale* F.H.Wigg.; Asteraceae LE 01063354 LE 01063387	Rus. *oduvanchik* Kar—	flowers	boiled with sugar	“honey”	1/2	3/1	2/1
		leaves	boiled or soaked	salad	1		
				soup	1		
			fresh	salad	3	4	
				soup			1
			soaked in salted water	salad			1
		roots	dried and roasted	coffee substitute	1	1	3
		sap	fresh	licked	/1	/1	
*Tussilago farfara* L.; Asteraceae LE 01063378	Rus. *mat’-i-machekha* Kar—	leaves	fresh	recreational tea			/1
*Alnus incana* (L.) Moench; Betulaceae LE 01063373	Rus. *ol’kha* Kar. *lepp*	wood	dried	smoking fish	14	5	10
				smoking meat	1		1
*Betula* spp.; Betulaceae LE 01063357	Rus. *bereza* Kar. *koivu*	buds	fresh	strong alcohol	1		
		leaves	fresh	recreational tea			/1
				salad	1		
				snack			/1
		sap	fermented	*kvass*	1/1	1	4
				*okroshka*	1	1	
				strong alcohol	1		1
			fresh	drink	9/14	7/8	5/16
			pasteurized	drink	2	1/1	2/1
*Barbarea vulgaris* R.Br.; Brassicaceae	Rus. *surepka* Kar—	aerial parts	fresh	cooking with fish		1	
				salad		1	
				soup		1	
*Bunias orientalis* L.; Brassicaceae	Rus. *sergibus*, *dikaia kapusta* Kar—	stems	fresh	snack			/1
*Lonicera caerulea* subsp. *pallasii* (Ledeb.) Browicz; Caprifoliaceae	Rus. *zhimolost’* Kar—	fruits	fresh	*mors*		/1	
				snack		/1	
*Stellaria media* (L.) Vill.; Caryophyllaceae LE 01063380 LE 01063381	Rus. *lapchatka*, *mokritsa* Kar—	aerial parts	fresh	salad	1	2	2
				soup		1	/1
*Juniperus communis* L.; Cupressaceae LE 01063493	Rus. *veres*, *mozhzhevel’nik* Kar—	fruits	dried	add to meat dishes		1	/1
				addition to tea		1	
		wood	dried	smoking fish		3	
*Equisetum arvense* L.; Equisetaceae	Rus. *stolbiki*, *khvoshch* Kar—	stems	fresh	snack			/1
*Arctostaphylos uva-ursi* (L.) Spreng.; Ericaceae LE 01063347	Rus. *toloknianka* Kar—	fruits	fresh	snack		/1	
*Empetrum nigrum* L.; Ericaceae	Rus. *vodianika*, *voronika*, *medvezh’ia iagoda*, *svinika* Kar. *variksen marja*	fruits	baked	berry porridge		/1	
			concentrated juice	drink	3	1	
			fresh	*kompot*	2	1	
				*mors*		1	
				snack	2/2	/3	/2
			juice	drink	1		
			soaked in cold water	food		/1	/1
*Vaccinium myrtillus* L.; Ericaceae LE 01063348	Rus. *chernika* Kar. *mušt’oi*, *must’oi*, *mussikka*	fruits	boiled with sugar	jam	7/2	8/2	9
			boiled without sugar	pies	2		
				snack	1		
			concentrated juice	*kompot*	1		
				drink	1		
				*kissel*	1		
			dried	*kompot*			1
				drink			/1
				*kissel*	1/5	1	1
				*mors*	1	/1	
				pies	1/1	1	
				recreational tea	1/2	/1	
				snack	/1	/1	
			fresh	*kompot*	1	2	2
				condiment for birch sap *kvass*			/1
				eaten with curd		1	
				eaten with milk		2	
				eaten with porridge		1	
				eaten with sugar		1	/1
				juice	1		
				*kissel*	4/3	7	3/2
				*mors*	4/2	3	2
				*mousse*	/1		
				pies	9/2	10/1	10/1
				snack	5/3	2	2/2
				*vareniki*			1
			frozen	*kompot*		1	
				eaten unfrozen	2	2	
				eaten with porridge		1	
				*kissel*	1		
				*mors*	1	1	
				pies	1	2	
			jam	moonshine			/1
				pies	2/1	2	3
			juice	drink	1		2
			oven-baked	dessert	/1		
			smashed with sugar	raw jam		1	
			steamed	jam		1	
				*kissel*	1/1		1
				*kompot*	1		
				pies		1	2
				snack		1	
		leaves	dried	recreational tea	1/2	1	/1
*Vaccinium oxycoccos* L.; Ericaceae LE 01063360	Rus. *kliukva*, *zhuravina* Kar. *garbalo*, *karpalo*	fruits	boiled with sugar	jam	4	4	3
				jelly	1		
				syrup			1
			fermented	strong alcohol		/1	
			fresh	*kompot*			1
				condiment for birch sap *kvass*			/1
				condiment for sauerkraut	5/1	7/4	4/3
				drink	1/1	/2	
				*kissel*	6/7	11/1	7/3
				liquor		/1	
				*mors*	15/1	12/1	11/1
				*mousse*	1	/2	
				pies	2/2	2	7
				recreational tea		1	
				snack	1	3	1
			frozen	eaten with sugar	1		1
				*kissel*	4	4	1
				*mors*	5	4/2	3/2
				pies	1		
				snack	2/1	1/1	
			jam	*kissel*	1		
				*mors*	1		
				pies	2/1	1	
			juice	addition to vodka			1
			smashed with sugar	raw jam	1	1	
			soaked in cold water	jelly	1		
				*kissel*	1	2	
				*mors*	1	1	/2
				pies	1		
			soaked in own juice	eaten with sugar			/2
*Vaccinium uliginosum* L.; Ericaceae LE 01063389	Rus. *golubika*, *gonobel’*, *gonobobel’* Kar. *juopukka*, *sinine marja*	fruits	boiled with sugar	jam	1/1	1/2	2/1
			dried	pies		/1	
			fresh	*kissel*	/2	/1	
				pies	/1	/1	
				recreational tea		1	
				snack	1/4	3/1	2/3
				wine			1
			jam	moonshine			1
			wine	wine	/1		
*Vaccinium vitis-idaea* L.; Ericaceae LE 01063375	Rus. *brusnika* Kar. *buolu*, *puola*, *puolakka*	fruits	baked	berry porridge		/1	
			boiled	*mousse*	/1		
			boiled with sugar	jam	6/1	8/1	5/2
				jelly		1	
			concentrated juice	drink	1		
			crumpled and boil with water, sugar and rye flour	berry soup *marjarokka*	/1		
			desiccated berries	pies	1		
			dried	recreational tea		1	
			fermented	strong alcohol		1/1	
				wine	1		
			fresh	coloring moonshine	/1		
				*kompot*			1
				condiment for birch sap *kvass*			/1
				condiment for sauerkraut	/1	1	
				drink		/1	
				eaten	1	1	
				eaten with oatmeal *kissel*	/1		
				eaten with *tolokno*	/2	3/2	2/1
				*kissel*	4/4	6	4/2
				*kompot*	1		1
				*mors*	12/2	11/1	9/1
				*mousse*	2/2		
				pies	12/4	14/2	13/3
				snack	1/1	3	1
				soup	1		
			frozen	*kompot*	1		
				eaten with bread		/1	
				eaten with sugar	1		1
				eaten with *tolokno*	/1	1	
				food	/1	1	1
				*kissel*	1/1	2	
				*mors*	3	1/1	2
				pies	1/2	1	2
				snack	1	/1	
			jam	pies	/1	1	1
			juice	drink	1		
				*mousse*	1		
				addition to vodka			1
			smashed in own juice	*mors*		/1	
				pies		/1	
			smashed with sugar	dessert		1	
				pies	1		/1
			soaked in cold water	*kompot*			1
				snack		1	1
				drink		/1	2
				eaten with bread and milk		/1	
				*kissel*	3	2	
				*mors*	2	2	1
				pies		2/1	
			soaked in own juice	drink	/1		
				eaten with sugar		1	/1
				food	1/2		
				jam			/1
				*kissel*	1/1		
				*mors*	1	1	/2
				pies	3/1	1	1/1
			soaked in syrup	*kissel*	1		
				pies	1		1
			soaked with sugar	drink		/1	
				food		/1	
			soaked with sugar and clover and salt	raw jam		1	
			steamed	pies		/1	
		leaves	dried	recreational tea	1/3	1/1	1/1
			fresh	*mors*		1	
*Trifolium hybridum* L.; Fabaceae	Rus. *klever* Kar—	flowers	fresh	sucked nectar	/1		
*Trifolium* sp. (incl. Trifolium pratense L.); Fabaceae LE 01063464 LE 01063494	Rus. *klever*, *krasnyi klever*, *kashka* Kar—	flowers	boiled with sugar	jam		1	
			dried	recreational tea	1	1	
			fresh	salad	1		
				snack	/1	/1	/4
				sucked nectar	/7	/6	/4
		leaves	fresh	salad	1		
*Vicia cracca* L.; Fabaceae LE 01063418	Rus. *dikaia vika* Kar—	fruits	fresh	snack			/1
*Quercus robur* L.; Fagaceae LE 01063338	Rus. *dub* Kar—	acorns	dried and roasted	coffee substitute		1	
		leaves	dried	recreational tea		1	
			fresh	condiment for cucumbers	/1	1	/1
				condiment for mushrooms	/1		/1
*Ribes nigrum* L.; Grossulariaceae LE 01063377	Rus. *chernaia smorodina* Kar. *viinimarja*, *mušta viinimarja*, *čiihi*	fruits	boiled with sugar	jam			/1
			fresh	snack		/1	/1
		leaves	fresh	condiment for mushrooms		1	1
				lemonade	1		
		leaves/shoots	dried	recreational tea	1/1	/1	1
*Hypericum* spp. (incl. *H. maculatum* Crantz and *H. perforatum* L.); Hypericaceae LE 01063492 LE 01063483 LE 01063538	Rus. *zveroboi* Kar. *kuzmanhattu*	aerial parts	dried	condiment		1	
				cooking with fish		1	
				recreational tea	1/2	5	/1
		root	fresh	strong alcohol	1		
*Mentha* sp.; Lamiaceae	Rus. *miata* Kar. *hajuheinä*	leaves	fresh	recreational tea	/1		
*Origanum vulgare* L.; Lamiaceae	Rus. *dushitsa* Kar—	aerial parts	dried	spice		2	
				recreational tea		2	
*Thymus subarcticus* Klokov & Desjat.-Shost.; LamiaceaeLE 01063500	Rus. *tim’ian*, *chabrets* Kar—	aerial parts	dried	spice		2	
				recreational tea		2	1
*Syringa vulgaris* L.; Oleaceae LE 01063339	Rus. *siren’* Kar—	flowers	boiled with sugar	jam	1		
*Epilobium angustifolium* L.; Onagraceae LE 01063353	Rus. *ivan-chai*, *kiprei*, *koporskii chai*, *koporka* Kar—	aerial parts	dried	recreational tea	2/4	2/2	4/9
			fermented	recreational tea	1/1	7/1	3
			smoked	recreational tea		1	
*Oxalis acetosella* L.; Oxalidaceae	Rus. *dikii shchavel’*, *zaiachii klever*, *zaiachii list*, *zaiach’ia trava*, *zaiach’i ushki*, *zaiach’ia kapust(k)a*, *zaiach’ia kislika*, *(zaiach’ia) kislitsa*, *lisichkin khleb* Kar—	flowers	fresh	snack		/2	/1
		leaves	fresh	snack	/9	1/8	1/11
				soup	/1		/1
*Picea x fennica* (Regel) Kom.; Pinaceae LE 01063512	Rus. *el’* Kar. *kuuzi*	cones	boiled with sugar	jam		1	
		needles	dried	recreational tea		1	
		resin	fresh	snack			/1
		wood	dried	smoking fish		1	
*Pinus sylvestris* L.; Pinaceae LE 01063372	Rus. *sosna* Kar. *pedäi*	buds	fresh	snack		/1	
		cones	boiled with sugar	jam		3	1
		needles	dried	recreational tea		1	
			fresh	snack		1	
		resin	fresh	snack			/3
		shoots	boiled into syrup and mixed with vodka	strong alcohol		2	
		wood	dried	add to flour			/1
*Linaria vulgaris* Mill.; Plantaginaceae LE 01063385	Rus. *l’nianka*, *iarutka* Kar—	flowers	fresh	snack		/1	/1
*Plantago* spp. (*P. major* L./*P. media* L.); Plantaginaceae LE 01063349	Rus. *podorozhnik* Kar—	leaves	fresh	salad	1		
				snack	/1		
		seeds	dried	condiment for bread		1	
*Elymus repens* (L.) Gould; Poaceae	Rus. *pyrei* Kar—	roots	fresh	salad	1		
				snack	/1		
			roasted	snack	/1		
		stems	fresh	snack			/1
*Phleum pratense* L.; Poaceae LE 01063344	Rus. *timofeevka* Kar—	stems	fresh	snack			/1
*Phragmites australis* (Cav.) Trin. ex Steud.; Poaceae	Rus. *trostnik ozernyi* Kar—	spring shoots	fresh	snack		1	
Poaceae	Rus. *trava* Kar—	stems	fresh	snack			/1
*Polygonum aviculare* L.; Polygonaceae LE 01063535 LE 01063533	Rus. *gorets ptichii* Kar—	aerial parts	fresh	salad		1	
*Rumex confertus* Willd.; Polygonaceae	Rus. *konskii shchavel’* Kar—	leaves	fresh	snack			/2
				soup			/1
*Rumex* spp. (*R. acetosa* L./*R. acetosella* L./Rumex longifolius DC./Rumex thyrsiflorus Fingers.); Polygonaceae LE 01063383 LE 01063540 LE 01063513 LE 01063479	Rus. *zaiach’ia kapusta*, *(lesnoi) shchavel’*, *kislika*, *kislitsa* Kar. *suoluheinä*	leaves	fresh	eaten fresh with sugar	/1		
				pies		/1	
				salad			/1
				snack	/9	2/4	/11
				soup	2/8	2/7	3/8
			salted	soup			1
*Alchemilla vulgaris* L.; Rosaceae	Rus. *manzhetka* Kar—	leaves	fresh	salad	1		
*Crataegus chlorocarpa* Lenn‚ & K.Koch; Rosaceae LE 01063507	Rus. *boiaryshnik* Kar—	fruits	dried	recreational tea		1	1
*Filipendula ulmaria* (L.) Maxim.; Rosaceae LE 01063341 LE 01063420	Rus. *tavolga*, *labaznik* Kar—	aerial parts	dried	recreational tea	3	1	
*Fragaria vesca* L.; Rosaceae LE 01063499	Rus. *zemlianika* Kar. *mandžoi*	fruits	boiled with sugar	jam	3	1	1/2
			dried	recreational tea		2	/1
			fresh	eaten fresh with sugar			/1
				*kissel*			/1
				snack	4/3	8	5/4
			jam	drink			/1
		leaves	dried	addition to tea		4	2
			fresh	snack		1	
*Malus* sp.; Rosaceae	Rus. *iablonia* Kar—	fruits	fresh	pies			1
*Prunus padus* L.; Rosaceae LE 01063366 LE 01063361	Rus. *cheremukha* Kar. *tuomi*, *tuomimarju*	fruits	boiled with sugar	jam			1/1
			dried	add to porridge	/1		
				food	/1		
				pies			/1
			fresh	*kompot*	1/1	1	
				*kissel*		1	
				pies		1	1/1
				snack	/9	1/1	/6
				wine			/1
*Rosa* spp. (incl. *R. rugosa* Thunb.); Rosaceae LE 01063491	Rus. *shipovnik* Kar—	flowers/fruits	boiled with sugar	jam		2	
		fruits	dried, fresh	recreational tea	/2	4	1/1
			fresh	snack		/1	
*Rubus arcticus* L.; Rosaceae	Rus. *tsar’-iagoda*, *kniazhenika*, *polenika*, *kumanika*, *ezhevika* Kar. *hepokka*	fruits	boiled with sugar	jam	1		
			fresh	snack	/1	2	
			frozen	snack	1		
			soaked under weight	snack	1		
		fruits, twigs	fresh	*kissel*	/1		
*Rubus chamaemorus* L.; Rosaceae LE 01063345	Rus. *moroshka*, *rokhletsy* Kar. *muuroi*, *muur’oi*, *hillo*, *lakka*, *šl’uboi*	fruits	boiled with sugar	jam	10/1	8/3	3/1
			dried	recreational tea		2	
			fresh	*kompot*	1	1	
				dessert with sugar	1		/1
				eaten		/1	1
				*kissel*		/1	
				*mors*	1		
				pies	3/1	5	2/1
				snack	5/2	7/2	4
			frozen	pies	1	2	
				snack	1	1	
				*mors*	1		
			frozen with sugar	eaten with ice-cream		1	
				smoothie		1	
				snack			
			jam	drink			/1
				moonshine			2
				pies	2		
				tincture			
			poured with sugar	raw jam		1	
			preserved in bog	eaten in own juice			/1
			pressed with vodka	pies		/1	
			smashed, poured with vodka	food			2
			soaked in own juice	food	1/1	1	2/1
				*kissel*			/1
				*mors*			1
				pies			1
				snack	2	1	
			soaked in syrup	syrup		1	
			soaked in water	eaten with sour cream	/1		
				food	/1	1/1	/1
				jam			1
				pies	/1		
		leaves	dried	recreational tea		1	
		sepals	dried	recreational tea	3	1	1
*Rubus idaeus* L.; Rosaceae LE 01063355	Rus. *malina* Kar. *vagoi*, *vavoi*, *vavarno*, *malina*	fruits	boiled with sugar	jam	7/1	10/1	9/2
			boiled with sugar	*kissel*	/1		
			dried	recreational tea	1/1		/1
			fermented	strong alcohol		1	
			fresh	kompot	1	1	1
				eaten fresh with sugar			/1
				eaten with oatmeal *kissel*	/1		
				*kissel*	/1		
				*mors*	1		
				pies	5/1	1	3/1
				recreational tea		1	
				snack	4	1/2	2/2
			frozen	eaten with sugar			1
				food	1	1	
				*mors*	1		
			jam	drink			/1
				drink after sauna	/1		
				pies	/2		1/1
			juice	drink	1		
		leaves	dried	recreational tea	/2	2/2	2
			fresh	condiment for cucumbers	1/1		
				recreational tea			1/1
		twigs with berries and leaves	dried	recreational tea	3/6	2/1	6
*Rubus nessensis* Hall; Rosaceae	Rus. *ezhevika*, *kumanika* Kar—	fruits	fresh	snack	/5	3	/1
*Rubus saxatilis* L.; Rosaceae	Rus. *kostianika* Kar. *juomoi*	fruits	boiled with sugar	jam	1		
			fresh	recreational tea		1	
				snack	/4	2/1	2/2
*Sorbus aucuparia* L.; Rosaceae LE 01063371	Rus. *riabina* Kar. *pihl’u*	branch with fruits	soaked in water	food			/1
			frozen	eaten frozen		1/1	
		fruits	boiled with sugar	jam		1/2	1/2
			dried	*kompot*		1	
				recreational tea		3/1	2/2
				snack	/2	1	/1
			fermented	wine		2/1	
			fresh	*kompot*			/2
				drink	1		
				snack	/1	1/1	
			frozen	raw jam	/1		1
				recreational tea			/1
			jam	moonshine			/1
				*mors*		/1	/1
		wood	dried	smoking fish	1	1	
*Populus tremula* L.; Salicaceae	Rus. *osina* Kar. *pedäi*	wood	dried	smoking fish	4	1	2
				smoking meat		/1	
*Urtica dioica* L.; Urticaceae LE 01063363	Rus. *krapiva* Kar. *šiiloi, žiiloi*	young aerial parts	dried	sandwich		1	
				soup	1		
			fresh	cooking with fish		1	
				salad		1	
				soup	4/7	7/5	4/11
*Viburnum opulus* L.; Viburnaceae	Rus. *kalina* Kar—	fruits	boiled	snack		1	
			boiled with sugar	jam	1		
				juice	1		
			fresh	mixed with sugar		/1	
				*mors*		1	
				snack	1		
				strong alcohol		1	1
			frozen	snack		/1	1
			steamed	preserve			/1

KAR: Karelian; RUS: Russian; OTH: other ethnic groups.

**Table 3:** Historical uses of wild plants in Karelia.

Plant Taxa	Local Name	Used Part	Preparation	Use	KAR	RUS	Source
*Sagittaria sagittifolia* L.; Alismataceae	Rus. *strelolist*	root	-	additive to flour	-	-	[17]
*Allium* sp.; Amaryllidaceae	Rus. *dikii luk*	-	-	-	X	X	[47] [58]
*Heracleum* sp.; Apiaceae	Rus. *borshch*		boil	cooling drink		X	[59]
*Calla palustris* L; Araceae	Rus. *zhitnitsa*, *khlebnik*, *khlebnitsa*, Kar.* vehka*	rhizomes	-	additive to flour	X		[45]
					-	-	[17]
*Cichorium intybus* L.; Asteraceae	Rus. *tsikorii*	roots	fry	coffee substitute	X	X	[50] [49]
*Betula* spp.; Betulaceae	Rus. *bereza*	sawdust	-	additive to flour	X		[52]
		bark	-	additive to flour	X		[52]
		sap	-	moonshine	X		[53]
*Butomus umbellatus* L.; Butomaceae	Rus. *susak*	roots	-	additive to flour	-	-	[17]
*Pteridium aquilinum* (L.) Kuhn; Dennstaedtiaceae	Rus. *paporotnik*	shoots	fresh	snack	X		[52]
			boil	soup	X		[52]
*Empetrum nigrum* L.; Ericaceae	Rus. *voronika*, Kar. *kuarnehuš*, *puarnahus*	fruits	-	-		X	[60]
			crush in barrel	-	X		[53]
*Vaccinium myrtillus* L.; Ericaceae	Rus. *chernika*, Kar. mussikka, mušt’oi, must’oi	fruits	-	- - - - soup *marjarokka*	X X X	X X	[44] [60] [61] [51] [21]
			dry	pies	X		[59]
				-	X		[50]
			boil	pies	X		[21]
				-	X		[53]
			fresh	pies	X	X	[49] [21]
				snack		X	[49]
				jam	X		[21]
				smashed with sugar	X		[21]
				eaten with milk	X		[21]
				drink	X		[21]
				sandwich	X		[21]
				-	X		[50]
						X	[62]
*Vaccinium oxycoccos* L.; Ericaceae	Rus. *kliukva*, Kar. *karpalo*, *garbal*, *guarbalo*	fruits	-	-	X		[47]
						X	[60]
					X		[59]
					X		[51]
				drink	X		[50]
				pies		X	[22]
						X	[49]
			fresh	*kissel*	X		[53]
				dessert	X		[53]
				jam	X		[21]
				mousse	X		[21]
				*kvass*	X		[21]
				condiment for sauerkraut		X	[49]
				-	X	X	[50] [62]
			soak	garnish	X		[21]
				salad	X		[21]
				*kissel*	X		[21]
				*mors*	X		[21]
				condiment for sauerkraut	X		[21]
			smash or mince with sugar	*kissel*	X		[21]
				*mors*	X		[21]
				pies	X		[21]
				dessert	X		[21]
*Vaccinium uliginosum* L.; Ericaceae	Rus. *golubika*, Kar. *juopukko*, *düobukko*, *juamoi*	fruits	boil	eat	X		[53]
			fresh	-		X	[62]
			-	drink	X		[53]
				soup *marjarokka*	X		[21]
				-	X		[59]
*Vaccinium vitis-idaea* L.; Ericaceae	Rus. *brusnika*; Kar. *puola*, *buol*, *buolu*	fruits	-	- - - - - - -	X X X X	X X X	[47] [63] [61] [60] [64] [51] [62]
				ritual dish	X		[65]
				*kissel*		X	[49]
				pies	X	X	[49] [21]
				soup *marjarokka*	X		[22]
					X		[21]
				tea substitute	X		[22]
				bake in turnip	X		[21]
			crush in barrel	eat with boiled turnip	X		[21]
				eat with *tolokno*	X		[21]
				-	X		[53]
				-	X		[50]
			freeze	dessert	X		[53]
				pies	X		[53]
				eat with *tolokno*	X		[53]
				eat with rye flour	X		[53]
			soak	eat with sweetened water	X		[59]
				cowberry water		X	[49]
				-	X		[53]
				pies	X		[21]
				garnish	X		[21]
				*kissel*	X		[21]
				*mors*	X		[21]
				salad	X		[21]
			fresh	eaten with *tolokno*	X		[66]
					X		[22]
				jam	X		[21]
				fruit puree	X		[21]
				eaten with milk	X		[21]
				pies	X		[21]
				condiment for apples	X		[50]
		leaves	dry	tea substitute	X	X	[49] [53]
*Trifolium* sp.; Fabaceae	Rus. *klever*	flowers	dry	additive to flour	X		[52]
*Ribes nigrum* L.; Grossulariaceae	Rus. *smorodina*	leaves	dry	tea substitute	X	X	[49] [52]
*Hypericum* spp.; Hypericaceae	Rus. *zveroboi*	-	dry	tea substitute	X	X	[49] [52]
		flowers	dry	tea substitute	X		[53]
*Iris pseudacorus* L.; Iridaceae	Rus. *iris*	rhizomes	-	additive to flour	-	-	[67]
*Mentha* sp.; Lamiaceae	Rus. *miata*	aerial parts	dry	tea substitute	X	X	[49] [52]
*Thymus subarcticus* Klokov & Desjat.-Shost.; Lamiaceae	Rus. *bogoroditska ia trava*	aerial parts	dry	tea substitute		X	[49]
*Nuphar luteum* (L.) Sm.; Nymphaeaceae	Rus. *odolen’ koren’*	rhizomes	-	additive to flour	-	-	[67]
*Nymphaea alba* L.; Nymphaeaceae	Rus. *odolen’ koren’*	rhizomes	-	additive to flour	-	-	[67]
*Cetraria islandica* (L.) Ach.; Parmeliaceae	Rus. *mokh*	thallus	soak in liquor, rinse and dry *	additive to flour	-	-	[68]
*Pinus sylvestris* L.; Pinaceae	Rus. *sosna*	cambium	dry, bake, and grind	additive to flour	X		[45]
				additive to fish soup	X		[45]
			-	additive to flour	X		[63]
					X		[15]
						X	[69]
					X		[64]
					X		[70]
					X		[52]
					X		[53]
			fresh	snack		X	[59]
*Glyceria fluitans* (L.) R.Br.; Poaceae	Rus. *mannik*	seeds	-	additive to flour			[67]
*Rumex* sp.; Polygonaceae	Rus. *shchavel’*, Kar. *suoluheinä*	leaves	fresh	snack		X	[59]
				pies	X		[21]
			boil	soup *suoluheinusuupu*, *čuokoisuupu*	X		[66]
					X		[21]
			-	-	X		[47]
*Filipendula ulmaria* (L.) Maxim.; Rosaceae	Rus. *tavolga*	rhizomes	-	additive to flour			[17]
*Fragaria vesca* L.; Rosaceae	Rus. zemlianika	fruits	fresh	sandwich	X		[21]
				-		X	[62]
			-	-	X		[71]
*Prunus padus* L.; Rosaceae	Rus. *cheremukha*	fruits	fresh	snack		X	[49]
*Rosa* sp.; Rosaceae	Rus. *shipovnik*	fruits	fresh	tea substitute	X		[21]
			dry	tea substitute	X		[21]
*Rubus chamaemorus* L.; Rosaceae	Rus. *moroshka*, *hillo*, *muuroi*	fruits	soak	-	X X X	X X X	[46] [47] [53] [49] [21] [62]
			fresh	snack	X		[47]
				jam	X		[21]
				-		X	[62]
			fermented	eat with sweetened water	X		[59]
			-	-	X X X X X	X	[60] [61] [71] [72] [64] [51]
*Rubus idaeus* L.; Rosaceae	Rus. *malina*, Kar. *malina*, *vagarm*, *vavarno*	fruits	boil	-	X		[53]
			fresh	snack		X	[49]
				jam	X		[21]
				smashed with sugar	X		[21]
				eaten with milk	X		[21]
				sandwich	X		[21]
				-		X	[62]
			-	-	X X	X	[61] [71] [51]
				soup *marjarokka*	X		[21]
		leaves and stems	dry	tea substitute	X X		[49] [53]
		leaves	ferment and dry	tea substitute	X		[51]
			dry	tea substitute			[52]
*Sorbus aucuparia* L.; Rosaceae	Rus. *riabina*	fruits	fresh	snack		X	[49]
				jam	X		[21]
*Typha latifolia* L., *T. angustifolia* L.; Typhaceae	Rus. *chakan*, *rogoz*, *khlebnik*, *kuga*, *sitnik*, *pshenichka*, *boby*	young stems	fresh	snack			[17]
		rhizomes	bake	additive to flour			[17]
*Urtica* sp.; Urticaceae	Rus. *krapiva*, Kar. *šiiloi*	aerial parts	boil	soup *čiiloirokku*	X		[21]
*Viburnum opulus* L.; Viburnaceae	Rus. *kalina*	fruits	steam	*kissel*		X	[49]
“roots”	Rus. *koreshki*	roots		snack for children		X	[59]
moss	Rus. *mokh*	aerial parts	dry	additive to flour	X		[53]

* Recommended use. Information about actual use was not found. ‘X’ reflects the plant use record.

## References

[1] Pieroni A., Sõukand R., Amin H.I.M., Zahir H., Kukk T. (2018). Celebrating multi-religious co-existence in central Kurdistan: The bio-culturally diverse traditional gathering of wild vegetables among Yazidis, Assyrians, and Muslim Kurds. Hum. Ecol..

[2] Quave C.L., Pieroni A. (2015). A reservoir of ethnobotanical knowledge informs resilient food security and health strategies in the Balkans. Nat. Plants.

[3] Pieroni A., Sõukand R. (2019). Ethnic and religious affiliations affect traditional wild plant foraging in central Azerbaijan. Genet. Resour. Crop Evol..

[4] Karst A. (2010). Conservation Value of the North American Boreal Forest from an Ethnobotanical Perspective.

[5] Pouta E., Sievänen T., Neuvonen M. (2006). Recreational wild berry picking in Finland—Reflection of a rural lifestyle. Soc. Nat. Resour..

[6] Caldwell M.L. (2007). Feeding the body and nourishing the soul: Natural foods in postsocialist Russia. Food Cult. Soc..

[7] Reyes-García V., Menendez-Baceta G., Aceituno-Mata L., Acosta-Naranjo R., Calvet-Mir L., Domínguez P., Garnatje T., Gómez-Baggethun E., Molina-Bustamante M., Molina M. (2015). From famine foods to delicatessen: Interpreting trends in the use of wild edible plants through cultural ecosystem services. Ecol. Econ..

[8] Shikov A.N., Tsitsilin A.N., Pozharitskaya O.N., Makarov V.G., Heinrich M. (2017). Traditional and current food use of wild plants listed in the russian pharmacopoeia. Front. Pharm..

[9] Lebedeva T.P. (1999). Materials for the ethnobotany of Finno-Ugric population of the east of Leningrad oblast’. Vestn. St. Peterbg. Univ..

[10] Lebedeva T.P., Tkachenko K.G. (2017). Peculiarities of the use of some taxa in traditional practices by the ethnic minorities of the Russian North. Traditsionnaia Meditsina. Nauchno Prakt. Zhurnal.

[11] Stryamets N., Elbakidze M., Ceuterick M., Angelstam P., Axelsson R. (2015). From economic survival to recreation: Contemporary uses of wild food and medicine in rural Sweden, Ukraine and NW Russia. J. Ethnobiol. Ethnomed..

[12] Jernigan K., Belichenko O., Kolosova V., Orr D. (2017). Naukan ethnobotany in post-Soviet times: Lost edibles and new medicinals. J. Ethnobiol. Ethnomed..

[13] Jernigan K., Belichenko O., Kolosova V., Orr D., Pupynina M. (2019). Gathering “mouse roots,” among the naukan and chukchi of the russian far east. Ethnobiol. Lett..

[14] Potanin G.N. (1876). Nikolsky uezd and its dwellers. Drevniaia Novaia Ross.

[15] Engel’gardt A.P. (1897). Russian North: Travel Notes.

[16] Klepikov S.A. (1920). Russian Peasants’ Diet.

[17] Gudkov A.G. (2006). Diet of the peasants of the Russian North in the end of XVIII*—*First half of XIX century. Evropeiskii Sever Rossii: Traditsiia i Modernizatsionnye Protsessy.

[18] Lebedeva T.P., Tkachenko K.G. (2016). Peculiarities of the use of local flora plants as medicine and food by the Karelians and Russians. Traditsionnaia Meditsina. Nauchno Prakt. Zhurnal.

[19] Lebedeva T.P., Tkachenko K.G. (2017). Peculiarities of the use of Sphagnum and Betula spp. by the Finnic peoples. Biulleten Bot. Sada Inst..

[20] Bromlei I.V. (1977). Contemporary Ethnic Processes in the USSR.

[21] Nikol’skaia R.F. (1989). The Karelian cuisine.

[22] Klement’ev E.I. (1981). Material Cultire and Applied Arts of the Segozero Karelians at the End of XIX-Beginning of XX Century.

[23] Kravchenko A.V. (2007). An Outline of the Flora of Karelia: A Monograph.

[24] Ignatova N.M. (2011). Forced Labor of Special Settlers in Economy of the Russian North in the 1930–1950s.

[25] Blandov A.A., Balt T.V., Krivosheeva I.V. (2009). Soviet Newcomers at the Karelian Isthmus and in the Ladoga Karelia in 1940s. Choosing a Coexistence Strategy.

[26] Vavulinskaia L.I., Zaitseva N.G., Zakharova E.V. (2015). Forced Industrial and Agricultural Migration to Karelia and Change in the Ethnic Composition of the Republic (end of 1940–1950s).

[27] Okorotysheva A.L., Pomorzhanskaia A.A., Golubeva N.V. (2004). Population Size and Composition of the Republic of Karelia According to the Data of All-Russian Census 2002: A Statistical Abstract.

[28] Makarov G.N. (1966). The Karelian Language. Iazyki Narodov SSSR.

[29] Koivisto V., Palander M., Riionheimo H., Koivisto V. (2018). Border Karelian dialects: A diffuse variety of Karelian. On the Border of Language and Dialect.

[30] All-Russian Census Returns of 2010. https://www.gks.ru/free_doc/new_site/perepis2010/croc/results.html.

[31] Karjalainen H., Puura U., Grünthal R., Kovaleva S. (2013). Karelian Language in Russia. ELDIA Report Studying the (Sociolinguistic) Situation.

[32] All-Russian Census of 2002. http://www.perepis2002.ru/index.html?id=12.

[33] All-Russian Census of 2010. https://gks.ru/free_doc/new_site/perepis2010/croc/perepis_itogi1612.html.

[34] Tánczos O.M. (2015). Representations of Karelians and the Karelian language in Karelian and Russian local newspapers. J. Est. Finno Ugric Linguist..

[35] Knuuttila S.-R. University of Joensuu Revitalization in Olonets Karelian: Towards functional bilingualism. Proceedings of the Two or More Languages, University of Joensuu.

[36] Tánczos O. (2018). Multilingual practices and speaker attitudes: The case of Olonets Karelian. Multilingual Practices in Finno-Ugric Communities. Ural. Hels..

[37] Antonova N., Language Nest (2018). How to Revialize the Karelian Language. http://www.ug.ru/archive/76612.

[38] The Plant List. http://www.theplantlist.org/.

[39] Stevens P.F. (2017). Angiosperm Phylogeny Website. http://www.mobot.org/MOBOT/research/APweb/.

[40] ISE Code of Ethics (with 2008 Additions). http://www.ethnobiology.net/what-we-do/core-programs/ise-ethics-program/code-of-ethics/code-in-english/.

[41] Kalle R., Sõukand R. (2016). Current and remembered past uses of wild food plants in Saaremaa, Estonia: Changes in the context of unlearning debt. Econ. Bot..

[42] Sõukand R., Kalle R. (2016). Changes in the Use of Wild Food Plants in Estonia: 18th–21st Century.

[43] Mauri M., Elli T., Caviglia G., Uboldi G., Azzi M. RAWGraphs: A visualisation platform to create open outputs. Proceedings of the 12th Biannual Conference on Italian SIGCHI Chapter.

[44] Kochkurkina S.I., Spiridonov A.M., Dzhakson T.N. (1990). Written Communications about the Karelians.

[45] Derzhavin G.R., Pimenov V.V., Epshtein E.M. (1958). Daily report composed during the review of the governorate by the chief of Olonets vicariate Derzhavin 1785 July 19. Russkie Issledovateli Karelii (XVIII v.). Ocherki.

[46] Korablev S.P. (1853). Description of the Ethnography of Manners for the Town of Onega of Arkhangel’sk Governorate, with a Compilation of Onega Songs, and an Inventory of Words Characterizing Local Dialect.

[47] Maksimov S.V., Izdanie knigoprodavtsa D.E. (1859). A Year in the North.

[48] Maksimov S.V. (1890). A literature expedition. Rus. Mysl’.

[49] Loginov K.K. (1993). Material Culture and Productive-And-Everyday Magic in Russians of Zaonezhie].

[50] Krasnopol’skaia T.V., Orfinskii V.P. (1997). Suisar’ Village: History, Everyday Life, Culture.

[51] Orfinskii V.P. (2001). Iukkoguba Village and its Districts.

[52] Orfinskii V.P. (2008). History and Culture of Siamozer’e.

[53] Taroeva R.F. (1965). Material Culture of the Karelians.

[54] Kalle R., Sõukand R. (2013). Wild plants eaten in childhood: A retrospective of Estonia in the 1970s–1990s. Bot. J. Linn. Soc..

[55] Merkulova V.A. (1967). Sketches of the Russian folk Nomenclature of Plants. Herbs, Mushrooms. Berries.

[56] Antipin V.K., Tokarev P.N. (2016). Dinamics of yield of swamp cranberry crops. Proceedings of the Rol’ Nauki V Reshenii Problem Regiona I Strany: Fundamental’nye I Prikladnye Issledovaniia.

[57] Sõukand R., Mattalia G., Kolosova V., Stryamets N., Prakofjewa J., Belichenko O., Kuznetsova N., Minuzzi S., Keedus L., Prūse B. (2020). Inventing a herbal tradition: The complex roots of the current popularity of Epilobium angustifolium in Eastern Europe. J. Ethnopharmacol..

[58] Zh A.A. (1912). Kanin and Timan tundras. Izv. Arkhangel’skogo Obs. Izucheniia Rus. Sev..

[59] Kalinin I. (1911). Onega dwellers. Izv. Arkhangel’skogo Obs. Izucheniia Rus. Sev..

[60] Ostr E. (1910). Summer in Pomorie. Izv. Arkhangel’skogo Obs. Izucheniia Rus. Sev..

[61] Rogachev K. (1910). Pertozero schismatic monastery. Izv. Arkhangel’skogo Obs. Izucheniia Rus. Sev..

[62] Loginov K.K. (2006). Ethnolocal Group of Russians in Vodlozerie.

[63] Ivanov A. (1863). Povenets Karelians, their home and social life and legends. Olonetskie Gubernskie Vedom..

[64] V-ov A. (1913). About Karelia. Izv. Arkhangel’skogo Obs. Izucheniia Rus. Sev..

[65] Leskov N. (1893). Report on a trip to the Olonets province in summer of 1892. Zhivaia Starina.

[66] Kryshen’ M.A., Iliukha O.P. (2013). The family cauldron boils thicker: Information on everyday and festive food of Ludic Karelians in dialect speech samples of 1950–1956. Karel’skaia Sem’a Vo Vtoroi Polovine XIX—Nachale XXI V.: Etnokul’turnaia Traditsiia V Kontekste Sotsial’nykh Transformatsii.

[67] Nigel’ B. (1841). Economic review of the north of Russia. Zhurnal Minist. Gos. Imushchestv.

[68] (1841). About food consumption of Iceland moss. Zhurnal Minist. Gos. Imushchestv.

[69] Milov L.V. (1998). Great Russian Farmer and Special Aspects of the Russian Historical Process.

[70] Gromov Z. (1873). Questionnaire of the Russian geographical society for ethnographic description, filled in by a local priest at the Karelians of Rebola uezd. Olonetskie Gubernskie Vedom.

[71] Dokuchaev-Baskov K. (1912). Trade in Kargopol’ uyezd of Olonets governorate. Izv. Arkhangel’skogo Obs. Izucheniia Rus. Sev..

[72] Pinegin N. (1909). Ain islands. Izv. Arkhangel’skogo Obs. Izucheniia Rus. Sev..

[73] Svanberg I., Nelson M.C., Häkkinen A. (1992). Bone meal porridge, lichen soup, or mushroom bread: Acceptance or rejection of food propaganda in Northern Sweden in the 1860s. Just a Sack of Potatoes? Crisis Experiences in European Societies, Past and Present.

[74] B–kov M. (1913). The everyday life of a Karelian. Izv. Arkhangel’skogo Obs. Izucheniia Rus. Sev..

[75] Chernov V.N. (1952). Useful Plants of the Karelo-Finnish SSR.

[76] Kujola J. (1944). Dictionary of Ludic Dialects.

[77] Makarov G.N. (1990). Dictionary of the Karelian Language.

[78] Eliseev I.S., Zaitseva N.G. (2007). Contrastive-Onomasiological Dictionary of the Dialects of the Karelian, Veps and Saami Languages.

[79] Fedotova V.P., Boiko T.P. (2009). Dictionary of the North Karelian Dialects.

[80] Zaikov P.M., Novak I.P., Pellinen N.A., Arkhipova N.N., Karakina V.I., Lettieva G.E., Medvedeva T.I., Spitsyna M.A. (2015). Russian–North-Karelian dictionary.

[81] Boiko T.P., Markianova L.F. (2016). The Big Russian-Karelian Dictionary (Livvik dialect).

[82] Kurkina N. (2014). The market of food ingridients: Facts and perspectives. Khleboprodukty.

[83] Freidin M.Z., Vasil’ev V.V. (2010). Increasing the economic efficiency of the culture and harvesting of cowberry plants. Vestsi Natsyianal’nai Akad. Navuk Belarusi. Seryia Agrar. Navuk.

[84] Tugolukov V.A. (1969). Pathfinders Astride the Reindeers.

